# GIBBERELLIN PERCEPTION SENSOR 2 reveals genesis and role of cellular GA dynamics in light-regulated hypocotyl growth

**DOI:** 10.1093/plcell/koae198

**Published:** 2024-07-23

**Authors:** Jayne Griffiths, Annalisa Rizza, Bijun Tang, Wolf B Frommer, Alexander M Jones

**Affiliations:** Sainsbury Laboratory, University of Cambridge, Cambridge, CB2 1LR, UK; Sainsbury Laboratory, University of Cambridge, Cambridge, CB2 1LR, UK; Sainsbury Laboratory, University of Cambridge, Cambridge, CB2 1LR, UK; Heinrich Heine University, Institute for Molecular Physiology, 40225 Düsseldorf, Germany; Sainsbury Laboratory, University of Cambridge, Cambridge, CB2 1LR, UK

## Abstract

The phytohormone gibberellic acid (GA) is critical for environmentally sensitive plant development including germination, skotomorphogenesis, and flowering. The Förster resonance energy transfer biosensor GIBBERELLIN PERCEPTION SENSOR1, which permits single-cell GA measurements in vivo, has been used to observe a GA gradient correlated with cell length in dark-grown, but not light-grown, hypocotyls. We sought to understand how light signaling integrates into cellular GA regulation. Here, we show how the E3 ligase CONSTITUTIVE PHOTOMORPHOGENESIS1 (COP1) and transcription factor ELONGATED HYPOCOTYL 5 (HY5) play central roles in directing cellular GA distribution in skoto- and photomorphogenic hypocotyls, respectively. We demonstrate that the expression pattern of the GA biosynthetic enzyme gene *GA20ox1* is the key determinant of the GA gradient in dark-grown hypocotyls and is a target of COP1 signaling. We engineered a second generation GPS2 biosensor with improved orthogonality and reversibility. GPS2 revealed a previously undetectable cellular pattern of GA depletion during the transition to growth in the light. This GA depletion partly explains the resetting of hypocotyl growth dynamics during photomorphogenesis. Achieving cell-level resolution has revealed how GA distributions link environmental conditions with morphology and morphological plasticity. The GPS2 biosensor is an ideal tool for GA studies in many conditions, organs, and plant species.

## Teaser

A dynamic cellular gradient of gibberellin quantified using a Förster resonance energy transfer biosensor is required but not sufficient to pattern cell growth and seedling stem morphogenesis in darkness and light.

## Introduction

The cellular dynamics of growth regulatory phytohormones act as signal integration nodes that allow plant development to continually adjust to environmental conditions. Gibberellin (GA) hormones are required for cell and organ elongation in many developmental contexts, including several with implications for agricultural yields such as germination, budbreak, stem elongation, branching, and flowering (Gao and Chu 2020; Wang et al. 2021; Wu et al. 2021; Cheng et al. 2022; Liu et al. 2022). Early in the life-cycle of the model plant Arabidopsis (*Arabidopsis thaliana*), GA mediated organ elongation is highly plastic in response to the light environment. Pre-germination, light triggers GA accumulation that allows elongating radicles to emerge through seed coats, while post-germination, darkness triggers GA accumulation in elongating hypocotyls as part of the skotomorphogenesis program, i.e. etiolation, allowing for seedling emergence from soil. In contrast, post-germination illumination represses GA accumulation and hypocotyl elongation as part of photomorphogenesis or de-etiolation (Reid et al. 2002; Zhao et al. 2007; Alabadí et al. 2008; Weller et al. 2009).

We developed a genetically-encoded Förster resonance energy transfer (FRET) biosensor for GA, nuclear localized GIBBERELLIN PERCEPTION SENSOR 1 (nlsGPS1), that revealed cellular GA dynamics in skoto- and photomorphogenesis (Rizza et al. 2017). Interestingly, skotomorphogenic hypocotyls showed low GA levels in the apical hook grading to high GA levels in the sub-apical hypocotyl, a cellular dynamic that correlated with cell length patterns. However, it remained unclear how this cellular GA gradient arises, is reprogrammed for photomorphogenesis, and is quantitatively related to cell elongation.

A cellular gradient of a mobile hormone such as GA can emerge from a number of differentially regulated enzymatic steps including early biosynthetic steps leading to the mobile intermediate GA_12_, the penultimate step catalyzed by GIBBERELLIN 20-OXIDASEs (GA20ox), the final biosynthetic step catalyzed by GIBBERELLIN 3-OXIDASEs (GA3ox), and catabolic steps catalyzed by GIBBERELLIN 2-OXIDASES (GA2ox) or CYTOCHROME P450, FAMILY 714, SUBFAMILY A, POLYPEPTIDE 1s (CYP714A1s) (Yamaguchi et al. 2014; Hedden 2020). In addition to the etiolated hypocotyl gradient, nlsGPS1 also revealed a GA gradient correlated with cell length in the elongation zone of Arabidopsis root tips (Rizza et al. 2017). This finding controverted a computationally simulated root GA gradient based in part on the expression pattern of a GA20ox enzyme as this step was regarded as rate limiting (Band et al. 2012). Using biosensing, chemical and genetic perturbations, and further mathematical modeling, a key role for the GA3ox step in controlling the elongation zone GA gradient was recently revealed (Rizza et al. 2021). However, it remained unclear which steps are patterned to articulate cellular GA dynamics in the hypocotyl or how they are themselves regulated.

Etiolated hypocotyls express GA20ox1, GA20ox2, GA3ox1, GA2ox1, GA2ox2, GA2ox6, Ga2ox8, and GA2ox9 enzymes, and the expression of several enzymes changes dynamically in response to light during Arabidopsis de-etiolation (Sun et al. 2016). In de-etiolating pea seedlings, red, far-red, and blue light inhibit expression of GA3ox genes and lower GA levels within 2 to 4 h (Reid et al. 2002). We observed low GA levels without a clear GA gradient in light-grown Arabidopsis hypocotyls (Rizza et al. 2017), suggesting that elevated cellular GA levels in the sub-apical hypocotyl would be depleted during de-etiolation. Phytochrome inhibition of hypocotyl elongation is well known to involve lowered GA signaling (Kamiya and García-Martínez 1999; Hedden and Phillips 2000; García-Martinez and Gil 2001; Halliday and Fankhauser 2003; Vandenbussche et al. 2005). However, a double mutant in Arabidopsis red and far-red-sensing PHYTOCHROME A and PHYTOCHROME B (*phyA phyB*) expressing nlsGPS1 showed only modestly elevated GA levels in the light (Rizza et al. 2017). Blue light-sensing CRYPTOCHROMES (CRY1 and CRY2) are positive regulators of the catabolic *GA2ox* genes and negative regulators of the biosynthesis genes *GA20ox1* and *GA3ox1* in Arabidopsis (Zhao et al. 2007).

PHY and CRY signaling both antagonize the central skotomorphogenesis regulator CONSTITUTIVE PHOTOMORPHOGENESIS 1 (COP1), an E3 ubiquitin-ligase with myriad degradation targets (McNellis et al. 1994; Kim et al. 2017). Among these, ELONGATED HYPOCOTYL 5 (HY5) is a transcription factor playing a key role in promoting photomorphogenesis in the light (Koornneef et al. 1980; Xiao et al. 2021). On the other hand, transcription factors promoting skotomorphogenesis include a series of basic helix–loop–helix (bHLH) proteins PHYTOCHROME INTERACTING FACTORS (PIFs) (Leivar and Quail 2011) and brassinosteroid responsive BRI1-EMS SUPRESSOR1 (BES1) (Yin et al. 2002) and BRASSINAZOLE RESISTANT 1 (BZR1) (Wang et al. 2002). Although PIFs, BES1, and BZR1 are dependent on GA signaling for removal of antagonist DELLA proteins (de Lucas et al. 2008; Feng et al. 2008; Bai et al. 2012; Gallego-Bartolomé et al. 2012; Li et al. 2012; Oh et al. 2012; Wang et al. 2012; Oh et al. 2014), there is also evidence that they can promote GA accumulation in positive feedback loops acting in rapid elongation growth of the sub-apical hypocotyl (Rizza et al. 2017).

Here, we investigated the quantitative effects of the above enzymatic steps and light signaling components on the cellular GA dynamics of etiolated hypocotyls. In darkness, PIF and BR signaling influenced GA homeostasis but were not required for establishing the GA gradient while COP1 played a critical role in GA accumulation in the sub-apical hypocotyl. Although GA2ox catabolism contributed to limiting GA from the apical hook, we pinpointed spatial control over GA20ox1 as a key generator of the GA gradient as its expression correlated with cellular GA accumulation in wildtype and mutants and induction of GA20ox1 expression strongly raised GA levels across the etiolated hypocotyl. In the light, PHY and CRY signaling repressed GA accumulation, in part through HY5, and a *phyA phy B cry1 cry2* mutant restored the skotomorphogenic GA gradient. In order to observe the effects of de-etiolation on patterning of GA, we created a next-generation GPS2 biosensor that has improved reversibility over GPS1. GPS2 was also engineered to have increased orthogonality and showed a loss of GA hypersensitivity phenotypes previously observed in Arabidopsis expressing GPS1. During de-etiolation, GA remained low in the apical hook and depleted from the sub-apical hypocotyl, in keeping with lowered biosynthetic and increased catabolic gene expression. Interestingly, a *ga2ox* heptuple mutant overelongated during de-etiolation. Taken together, our results indicate that GA accumulation is necessary for growth of sub-apical cells but is not sufficient for setting cell length. Additionally, depletion of GA from the apical hook is not developmentally meaningful, but timely GA depletion during de-etiolation is important for growth arrest.

## Results

### The skotomorphogenesis agonists PIFs are required to maintain, but not generate GA gradient in dark-grown hypocotyls

We previously demonstrated a reduction of GA levels in a quadruple mutant (*pifq*) lacking 4 PIFs in the 3-d-old dark-grown hypocotyl (Rizza et al. 2017) and thus PIFs represented candidate light signaling components that could drive GA accumulation patterns in the etiolated hypocotyl. However, the *pifq* mutant is photomorphogenic with a relatively short hypocotyl and open cotyledons (Leivar et al. 2008) and thus GA regulation could be an indirect consequence of a broader fate switching. To further explore the effect of PIFs on hypocotyl development and GA distribution, we monitored the growth of *pifq* and Col-0 in the dark for 72 h after germination using an infrared camera.

**Figure 1.**
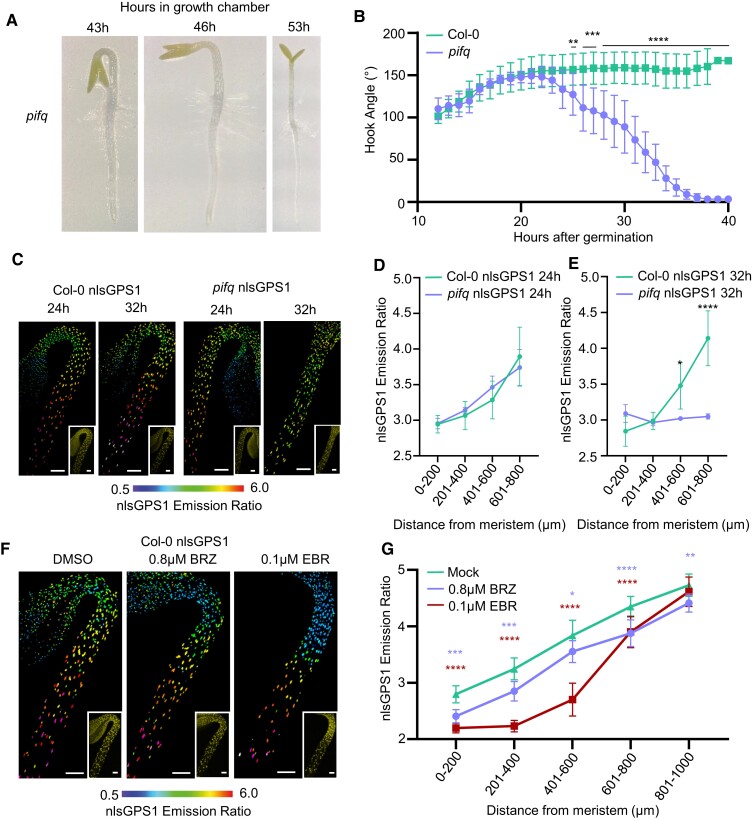
Skotomorphogenesis agonist PIFs are required to maintain, but not generate GA gradient in dark-grown hypocotyls. **A)** Representative images of *pifq* apical hook in dark-grown seedlings taken at 43, 46, and 53 h after start of 4 h germination light pulse. **B)** Apical hook angle in WT Col-0 vs *pifq* mutants with all seeds orientated horizontally to synchronize effects from gravity (see Materials and methods). Seedlings grown in the dark were imaged hourly in an infrared imaging chamber. 95% CI is shown. Repeated measures 2-way ANOVA followed by Tukey's post hoc test Col-0 *n* = 18 *pifq n* = 15 biologically independent hypocotyls (**P-value < 0.01, ***P-value < 0.001, ****P-value < 0.0001). **C)** nlsGPS1 emission ratios of dark-grown hypocotyls 24 and 32 h after germination. Representative images of emission ratios and YFP fluorescence (inset) are shown (additional images and color vision deficiency compatible LUT are shown in Supplementary Fig. S1). Scale bar = 100 *µ*m. **D)** Mean of mean nuclear emission ratios from 3 hypocotyls, binning by distance from the shoot apical meristem at 24 h after germination. Error bars = SD. Two-way ANOVA followed by a Holm–Šídák's post hoc test with no significant difference. **E)** Mean of mean nuclear emission ratios from 3 hypocotyls, binning by distance from the shoot apical meristem at 32 h after germination. Error bars = SD. Two-way ANOVA followed by a Holm–Šídák's post hoc test (*P-value < 0.05, ****P-value < 0.0001). **F)** nlsGPS1 emission ratios of 3 d dark-grown hypocotyls treated with 0.1 *µ*m epibrassinolide (EBR) or 0.8 *µ*m brassinozole (BRZ) for 24 h before imaging with DMSO treatment as mock. Representative images of emission ratios and YFP fluorescence (inset) are shown. Scale bars = 100 *µ*m (additional images and color vision deficiency compatible LUT are shown in Supplementary Fig. S2). **G)** Quantification of nlsGPS1 emission ratios in response to 24 h 0.1 *µ*m EBR or 0.8 *µ*m BRZ treatment. Mean of mean nuclear emission ratios from 8 hypocotyls, binning by distance from the shoot apical meristem. Error bars = SD. Two-way ANOVA followed by a Dunnett's post hoc test (*P-value < 0.05, **P-value < 0.01, ***P-value < 0.001, ****P-value < 0.0001). Colors correspond to treatment.

Apical hook development consists of 3 phases: formation, maintenance, and opening (Vandenbussche et al. 2010; Gallego-Bartolomé et al. 2011). Surprisingly, we observed the *pifq* mutant completing the formation phase and then opening without entering the maintenance phase (Fig. 1A and B). We imaged the upper hypocotyl (0 to ∼1,000 *µ*m from the shoot apical meristem) and analyzed GA levels in Col-0 nlsGPS1 and *pifq* nlsGPS1 at 32 h after germination when Col-0 was still in the maintenance phase and *pifq* was fully open. We observed that *pifq* seedlings have reduced GA levels in the sub-apical hypocotyl (∼500 to 1,000 *µ*m from shoot apical meristem; see Fig. 3B) and have no apical–basal GA gradient (Fig. 1C and E; Supplementary Fig. S1). However, at 24 h after germination when seedlings were at the end of formation phase, both Col-0 and *pifq* have an apical–basal GA gradient (Fig. 1C and D; Supplementary Fig. S1). The presence of and subsequent loss of the gradient in *pifq* indicates that PIFs are not required for the formation of the GA gradient but they are required for its maintenance.

### Brassinosteroid distribution does not explain GA gradient in dark-grown hypocotyls

Brassinosteroids (BRs) are known regulators of hypocotyl elongation and GA metabolism making them a candidate for controlling GA patterning. BRs have a dual regulatory role on GA levels promoting both GA biosynthesis and GA catabolism (Unterholzner et al. 2015; Albertos et al. 2022). We have established that the GA gradient is set up early in development. To investigate whether BR could be establishing this gradient, we first reduced BR levels by growing Col-0 nlsGPS1 in the presence of the BR biosynthesis inhibitor brassinazole (BRZ). To avoid germination effects, we germinated Col-0 nlsGPS1 on media and transferred seedlings to BRZ at 48 h after a germination light pulse. We observed an overall decrease in GA levels, however a clear apical–basal gradient of GA was maintained (Fig. 1F and G; Supplementary Fig. S2). Second, we increased BR levels by growing Col-0 nlsGPS1 in the presence of epibrassinolide (24-epiBL) and again saw an overall decrease of GA levels in the hypocotyl with persistence of the apical–basal gradient (Fig. 1F and G; Supplementary Fig. S2). These results support a complex role for brassinosteroids in the regulation of GA levels and indicate that BR levels are optimized in the plant to give high levels of GA that are required for rapid elongation in the dark. However, the persistence of the gradient under altered brassinosteroid levels indicates that brassinosteroids are not responsible for apical–basal patterning of GA levels.

### Skotomorphogenesis agonist COP1 is required for generating GA gradient and GA20ox1 and GA3ox1 expression in dark-grown hypocotyls

**Figure 2.**
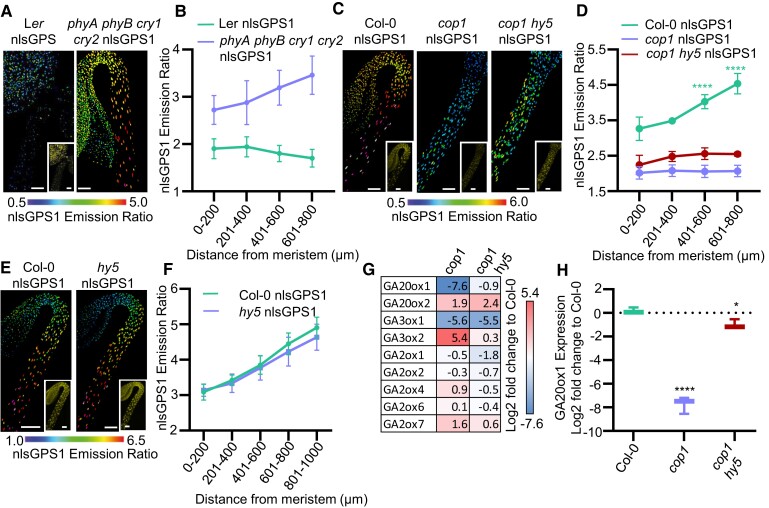
Skotomorphogenesis agonist COP1 is required for generating GA gradient and *GA20ox1* and *GA3ox1* expression in dark-grown hypocotyls. **A)** nlsGPS1 emission ratios of 3 d white light-grown hypocotyls of WT Ler and *phyA phyB cry1 cry2* mutant. Representative images of emission ratios are shown. Scale bar = 100 *µ*m. **B)** Mean of mean nuclear emission ratios from, binning by distance from the shoot apical meristem of 3 d dark-grown hypocotyls in WT Ler nlsGPS1 and *phyA phyB cry1 cry2* nlsGPS1 mutant. Error bars = SD. **C)** nlsGPS1 emission ratios of 3 d dark-grown hypocotyls of WT Col-0 and mutants *cop1-4* and *cop1-4 hy5-215*. Representative images of emission ratios and YFP fluorescence (inset) are shown (additional images and color vision deficiency compatible LUT are shown in Supplementary Fig. S3). Scale bar = 100 *µ*m. **D)** Mean of mean nuclear emission ratios from 4 hypocotyls, binning by distance from the shoot apical meristem of 3 d dark-grown hypocotyls for WT Col-0 and mutants *cop1-4* and *cop1-4 hy5-215*. Error bars = SD. Significance between distance windows comparing to the 0 to 200 *µ*m window 2-way ANOVA with a Dunnett's multiple comparisons test (****P-value < 0.0001). **E)** nlsGPS1 emission ratios of 3 d dark-grown hypocotyls of WT Col-0 and mutant *hy5-215*. Representative images of emission ratios and YFP fluorescence (inset) are shown (additional images and color vision deficiency compatible LUT are shown in Supplementary Fig. S4). Scale bar = 100 *µ*m. **F)** Mean of mean nuclear emission ratios from 5 hypocotyls, binning by distance from the shoot apical meristem. Error bars = SD. No significant difference by 2-way ANOVA with a Šídák's multiple comparisons test. **G)** Heat-map showing the fold change of relative expression of GA biosynthetic and catabolism genes in dark-grown hypocotyls of *cop1-4* and *cop1-4 hy5-215* mutants as compared to Col-0. The colors highlight log2 fold expression changes (red: increase; blue: decrease). Values were generated from 3 biological replicates of 30 hypocotyls measured in three technical repeats (for box and whisker (min–max) and statistical significance tests, see panel **H** and Supplementary Fig. S6). **H)***GA20ox1* expression in dark-grown hypocotyls. Log2 fold change from Col-0 for *cop1-4* and *cop1-4 hy5-215*. Box and whisker (min–max) show the median. A 1-way ANOVA was performed with a Dunnet's post hoc test for multiple comparisons (*P-value < 0.05, ****P-value < 0.0001).

Photoreceptors play key roles in both GA responsiveness and modulating GA levels. CRY1 and PHYA have been shown to redundantly regulate expression of GA metabolic enzymes and to lower GA levels in pea hypocotyls (Reid et al. 2002) and indeed we have previously shown that in Arabidopsis, the *phyA phyB* mutant has elevated levels of GA in the light (Rizza et al. 2017). CRYs are positive regulators of catabolic *GA2ox* genes and negative regulators of the biosynthesis genes *GA20ox1* and *GA3ox1* (Zhao et al. 2007). The quadruple *phyA phyB cry1 cry2* mutant grown under white light produces a hypocotyl similar in length to dark-grown wildtype plants (Mazzella et al. 2001). To further investigate the role of the photoreceptors in establishing the GA gradient, we created a *phyA phyB cry1 cry2* nlsGPS1 biosensor line. Under white light, photomorphogenesis is severely delayed in this line and we observed elevated levels of GA forming an apical–basal gradient (Fig. 2A and B). As PHYs and CRYs act to reduce GA levels in the light, we explored downstream targets of both CRY and PHY that act to establish the gradient in the dark.

The COP1 E3 ligase is inactivated by both photoreceptors and promotes skotomorphogenesis. As COP1 is required to maintain high levels of GA in dark-grown hypocotyls (Weller et al. 2009; Blanco-Touriñán et al. 2020), we explored the possibility that COP1 establishes the spatial distribution of GA. Using *cop1-4* nlsGPS1, we confirmed that GA levels are lower than WT and found that the apical–basal gradient is abrogated in dark-grown seedlings (Fig. 2C and D; Supplementary Fig. S3). A primary target of COP1 is the transcription factor HY5, a positive regulator of photomorphogenesis, which accumulates in dark-grown *cop1-4* seedlings (Osterlund et al. 2000). As in pea, the *hy5* mutation in the *cop1-4* background partially rescues both the phenotype and the GA levels of *cop1-4* in Arabidopsis (Fig. 2C; Supplementary Fig. S3) (Weller et al. 2009). However, using nlsGPS1, we observed that this rescue does not extend to the patterning of GA (Fig. 2D).

We explored the possibility that localized HY5 activity in the dark could repress GA accumulation in apical hooks but observed no effect on GA distribution in a *hy5-215* nlsGPS1 line (Fig. 2E and F; Supplementary Fig. S4). As expected, both the *cop1-4* and *cop1-4 hy5-215* double mutant showed rapid de-etiolation (Supplementary Fig. S5), unlike the *pifq* mutant that formed but failed to maintain an apical hook and GA gradient (Fig. 1B; Supplementary Fig. S5), suggesting that the GA gradient is linked with etiolation signaling mediated by COP1. Together, these results indicate that COP1 functions in part to prevent HY5 from repressing GA accumulation, though this function alone does not explain the central role for COP1 in gating the GA gradient.

GA biosynthetic enzymes are expressed in a tissue specific manner (Mitchum et al. 2006; Plackett et al. 2012) and the complexity of GA metabolism provides many candidate biochemical regulators of the hypocotyl GA gradient. We previously showed the key regulatory steps controlling a GA gradient in light-grown roots vary along the apical–basal axis (Rizza et al. 2021). In order to identify the specific GA metabolism targets of the COP1/HY5 pathway, we carried out RT-qPCR on 3-d-old dark-grown hypocotyls of WT, *cop1-4*, and *cop1-4 hy5-215* (Fig. 2G; Supplementary Fig. S6). We found that the genes involved in biosynthesis displayed greater changes than those involved in catabolism (Fig. 2G; Supplementary Fig. S6) with *GA20ox1* and *GA3ox1* being candidates for generating the GA gradient as both were strongly downregulated in *cop1-4* dark-grown hypocotyls. Interestingly, the expression of *GA20ox1* but not *GA3ox1* was partially rescued in the *cop1-4 hy5-215* mutant (Fig. 2G and H; Supplementary Fig. S6), consistent with a partial rescue of GA levels (Fig. 2C and D). The lack of a restored GA gradient in *cop1-4 hy5-215* could be an indication that although *GA20ox1* expression increased, patterned *GA20ox1* expression requires COP1-mediated etiolation signaling. Despite low GA levels in *cop1-4*, *GA20ox2* and *GA3ox2* were upregulated, potentially due to feedback regulation from lowered GA levels (Rieu et al. 2008) or, in the case of *GA3ox2*, upregulation via HY5 as the overexpression phenotype was rescued in the *cop1-4 hy5-215* mutant (Fig. 2G, Supplementary Fig. S6).

### GA20ox1 and GA3ox1 expression is associated with GA gradient

**Figure 3.**
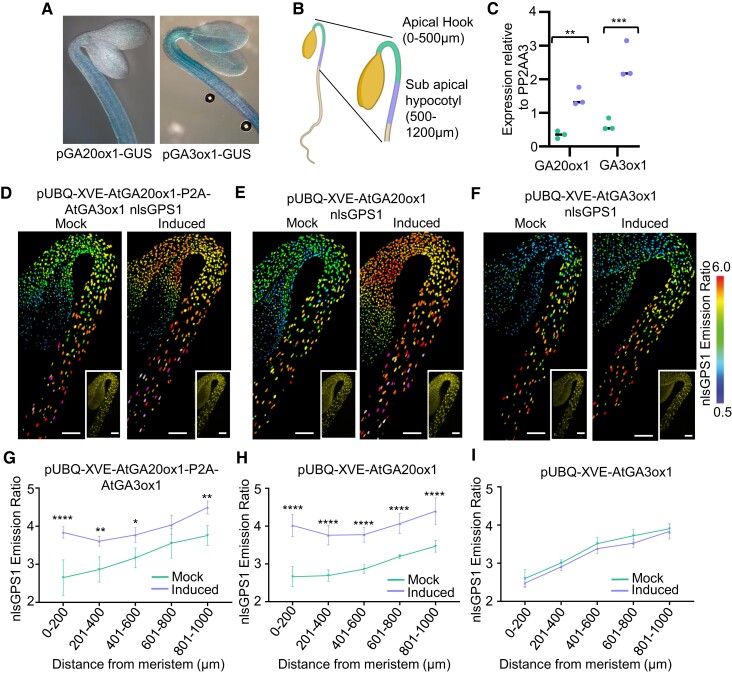
*GA3ox1* and *GA20ox1* expression is associated with GA gradient but only *GA20ox1* expression is rate limiting in dark-grown hypocotyls. **A)** Three-day-old dark-grown hypocotyls expressing AtGA20ox1 gene promoter in fusion with the GUS reporter and expressing AtGA3ox1 gene promoter in fusion with the GUS reporter. Lines are from Plackett et al. and Mitchum et al. pGA20ox1GUS is a translation fusion (TL) line and pGA3ox1GUS is a transcriptional fusion (TC) line. **B)** Schematic diagram showing the position of the apical hook and the sub-apical hypocotyl dissected for RT-qPCR analysis. Adapted from “Seedling 1”, by BioRender.com (2023). Retrieved from https://app.biorender.com/biorender-templates. **C)** Relative expression changes of *GA3ox1* and *GA20ox1* in the apical hook and sub-apical hypocotyl to control gene PP2AA3. Plants were grown for 3 d in the dark and gene expression analyzed by RT-qPCR. The data points and median of 3 biological replicates are plotted. Two-way ANOVA with a Šídák's post hoc test (**P-value < 0.01, ***P-value < 0.001). **D to F)** nlsGPS1 emission ratios of 3 d dark-grown hypocotyls β-estradiol inducible GA enzyme transgenic lines 48 h after induction with 2.5 *µ*m 17-β-estradiol (induced) or with 0.1% DMSO mock induction (mock). Representative images of emission ratios and YFP fluorescence (inset) are shown (additional images and color vision deficiency compatible LUT are shown in Supplementary Fig. S7). Scale bar = 100 *µ*m. **G to I)** Mean of mean nuclear emission ratios from 4 hypocotyls, binning by distance from the shoot apical meristem of 3 d dark-grown hypocotyls. β-Estradiol inducible GA enzyme transgenic lines 48 h after induction with 2.5 *µ*m 17-β-estradiol (induced) or with 0.1% DMSO mock induction (mock). Error bars = SD. Two-way ANOVA with a Šídák's multiple comparisons post hoc test for multiple comparisons between mock and induced (*P-value < 0.05, **P-value < 0.01, ****P-value < 0.0001).

To determine if differential expression of *GA20ox1* and *GA3ox1* is involved in establishing the GA gradient, we mapped the expression pattern of these 2 enzymes using pGA3ox1-TC-GUS and pGA20ox1-TL-GUS reporters (Mitchum et al. 2006; Plackett et al. 2012). In etiolated hypocotyls, expression of the 2 biosynthetic genes is high in the sub-apical hypocotyl and limited in the apical hook (Fig. 3A). To validate the expression observed using the reporter lines, RT-qPCR was carried out in dissected regions defined as the apical hook and the sub-apical hypocotyl (0 to 500 *µ*m and 500 to 1,200 *µ*m from the shoot apical meristem, respectively, Fig. 3B). The transcript levels of the 2 genes mirror the GUS staining with higher expression present in the sub-apical hypocotyl relative to the apical hook (Fig. 3C), and thus both are potentially responsible for generating the GA gradient.

### GA20ox1 is a key regulator of GA levels in the dark

In roots, GA20ox and GA3ox enzymatic steps have locally different roles with GA3ox being rate limiting in the elongation zone and both GA20ox and GA3ox being rate limiting in the root initials in the meristem (Rizza et al. 2021). To determine whether the expression of *GA20ox1* and *GA3ox1* may explain the GA gradient in the hypocotyl, we used a series of inducible overexpression lines previously used in roots (Rizza et al. 2021). Simultaneous β-estradiol induction of ubiquitous *AtGA20ox1* and *AtGA3ox1*, expressed as 1 transcript with the P2A ribosomal skipping sequence between the 2 genes, resulted in an increase of emission ratio along the hypocotyl with the greatest increase occurring in the apical hook (Fig. 3D and G; Supplementary Fig. S7). Lines that express *GA20ox1* or *GA3ox1* individually were used to further dissect the relationship between these GA biosynthetic enzymes and the GA gradient. Induction of *GA20ox1* increased the emission ratio of nlsGPS1 in a similar manner to that observed during simultaneous induction whereas induction of *GA3ox1* alone did not (Fig. 3E to I; Supplementary Fig. S7). As an increase of *GA20ox1* expression was sufficient to increase GA levels, *GA20ox1* is alone rate limiting for GA accumulation in the dark-grown hypocotyl, indicating that the expression pattern of this enzyme is a primary driver of the GA gradient here.

### Disruption of the GA gradient is not sufficient to abolish cell length patterning

**Figure 4.**
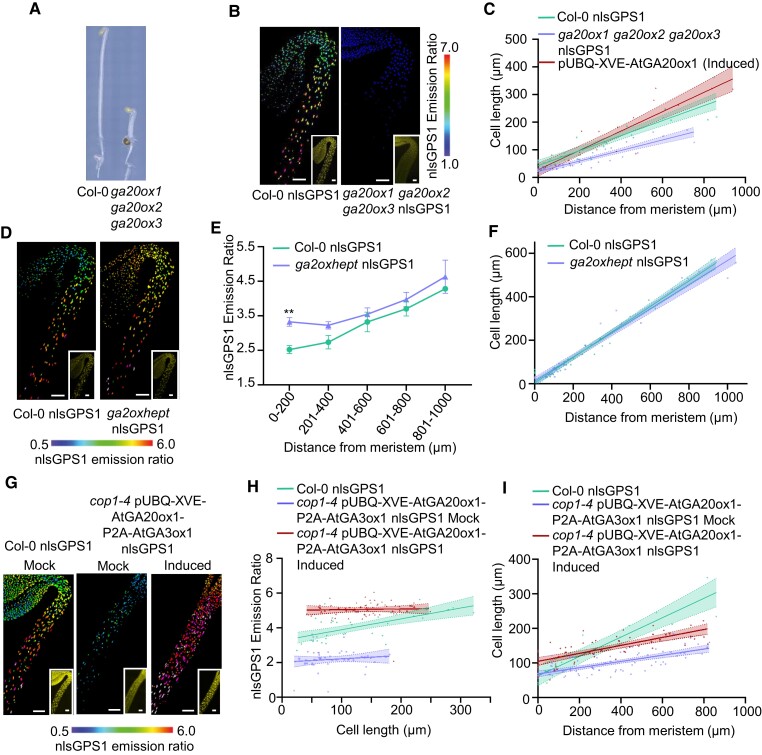
GA catabolism also plays a role in generating the GA gradient in dark-grown hypocotyls, but abolishing the GA gradient is not sufficient to abolish cell length patterning. **A)** Image of 3-d-old dark-grown *ga2ox1 ga2ox2 ga2ox3* mutant and Col-0 hypocotyls. **B)** Representative images of nlsGPS1emission ratios and YFP fluorescence (inset) 3-d-old dark-grown hypocotyls for WT Col-0 nlsGPS1 and *ga2ox1 ga2ox2 ga2ox3* nlsGPS1 are shown (additional images and color vision deficiency compatible LUT are shown in Supplementary Fig. S8). Scale bar = 100 *µ*m. **C)** Scatter plot showing cell length and distance from meristem for individual hypocotyl epidermal cells from the shoot apical meristem. Simple linear regression with 95% confidence intervals plotted. WT Col-0, *ga2ox1 ga2ox2 ga2ox3* mutant, and Col-0 pUBQ-XVE-GA20ox1 line induced with β-estradiol. β-Estradiol inducible GA enzyme transgenic line 48 h after induction with 2.5 *µ*m 17-β-estradiol (induced). For Col-0 hypocotyls, Pearson's correlation coefficient *r* = 0.92; *n* = 26 cells from 3 hypocotyls (1 cell file per hypocotyl); *ga2ox1 ga2ox2 ga2ox3 r* = 0.91, *n* = 35 cells from 3 hypocotyls; pUBQ-XVE-GA20ox1 (induced) *r* = 0.92 *n* = 27 cells from 3 hypocotyls. **D)** nlsGPS1 emission ratios of 3-d-old dark-grown hypocotyls for WT Col-0 and *ga2oxhept* mutant. Representative images of emission ratios and YFP fluorescence (inset) are shown (additional images and color vision deficiency compatible LUT are shown in Supplementary Fig. S9). Scale bar = 100 *µ*m. **E)** Mean of mean nuclear emission ratios from 3 hypocotyls, binning by distance from the shoot apical meristem of 3 d dark-grown hypocotyls of WT Col-0 and mutant *ga2oxhept*. Error bars = SD. Two-way ANOVA with a Šídák's post hoc test (**P-value < 0.01). **F)** Scatter plot showing protruding cell length and distance from meristem for individual hypocotyl epidermal cells from the shoot apical meristem. Simple linear regression with 95% confidence intervals plotted. For Col-0 hypocotyls, Pearson's correlation coefficient *r* = 0.9849; *n* = 31 cells from 3 hypocotyls (1 cell file per hypocotyl); *ga2oxhept r* = 0.9808, *n* = 29 cells from 3 hypocotyls. **G)** Representative images of emission ratios and YFP fluorescence (inset) are shown nlsGPS1 emission ratios of 3 d dark-grown hypocotyls for WT Col-0 nlsGPS1 and *cop1-4* pUBQ-XVE-AtGA20ox1-P2A-AtGA3ox1 nlsGPS1 (additional images and color vision deficiency compatible LUT are shown in Supplementary Fig. S11). Scale bar = 100 *µ*m. β-Estradiol inducible GA enzyme transgenic lines 48 h after induction with 2.5 *µ*m 17-β-estradiol (induced) or with 0.1% DMSO mock induction (mock). **H)** Scatter plot showing cell length and distance from meristem for individual hypocotyl epidermal cells from the shoot apical meristem. Simple linear regression with 95% confidence intervals plotted. For Col-0 nlGPS1 hypocotyls, Pearson's correlation coefficient *r* = 0.65; *n* = 36 cells from 3 hypocotyls. For *cop1-4* pUBQ-XVE-AtGA20ox1-P2A-AtGA3ox1 nlsGPS1, correlation coefficient *r* = 0.14 and 0.04; *n* = 46 and 65 cells for mock and induced, respectively. **I)** Scatter plot showing cell length and distance from meristem for individual hypocotyl epidermal cells from the shoot apical meristem. Simple linear regression with 95% confidence intervals plotted. For Col-0 nlGPS1 hypocotyls, Pearson's correlation coefficient *r* = 0.65; *n* = 36 cells from 3 hypocotyls. For *cop1-4* pUBQ-XVE-AtGA20ox1-P2A-AtGA3ox1 nlsGPS1, correlation coefficient *r* = 0.14 and 0.04; *n* = 46 and 65 cells for mock and induced, respectively.

The *ga20ox1 ga20ox2 ga20ox3* mutant has severe developmental defects associated with low levels of GA (Plackett et al. 2012) and we observed that hypocotyl elongation in the dark was dramatically reduced (Fig. 4A) as were the levels of GA (Fig. 4B; Supplementary Fig. S8). To explore the effect of spatial distribution of GA on cell size, we measured epidermal cell lengths in Col-0 nlsGPS1, *ga20ox1 ga20ox2 ga20ox3* nlsGPS1, and induced pUBQ-XVE-AtGA20ox1 lines. Cells in the apical hook are smaller than cells in the sub-apical hypocotyl (Gendreau et al. 1997) and this correlates with GA levels (Rizza et al. 2017).

As expected, we observed Col-0 cell lengths increasing with distance from meristem in hypocotyls of dark-grown seedlings (Fig. 4C). Interestingly, cell length remains patterned when the GA gradient is removed in *ga20ox1 ga20ox2 ga20ox3* or when GA levels are raised by inducing ubiquitous expression of *GA20ox1* (Fig. 4C). Sub-apical hypocotyl cells are shorter in *ga20ox1 ga20ox2 ga20ox3* indicating that the level of GA in these cells is important for elongation. However, higher levels of GA in the apical hook region with GA20ox1 overexpression do not correlate to longer cells suggesting that the relative depletion of GA is not important to keep these cells small. In light-grown hypocotyls, where higher GA levels do stimulate cell elongation, GA response was also shown to be positionally dependent along the apical–basal axis (Robinson et al. 2017). Our results indicate that the correlation between GA levels and cell length in etiolated hypocotyls, as in the root (Rizza et al. 2021), is not quantitatively causative in a simple dose-response relationship.

### GA catabolism also plays a role in generating the GA gradient in dark-grown hypocotyls

Nonetheless, it is intriguing that with the ubiquitous elevation of rate limiting *GA20ox1* expression, GA is slightly lower in the apical hook region compared with the sub-apical hypocotyl. The catabolic GA2ox family consists of 7 canonical members and 2 noncanonical members (Schomburg et al. 2003; Rieu et al. 2008; Lange et al. 2020). Expression data indicate that canonical *GA2ox1,2,4,6,7* and *8* are expressed in etiolating or de-etiolating hypocotyls (Sun et al. 2016). In order to determine whether these enzymes are in part responsible for the lowering of GA in the hook, we created a *ga2ox* heptuple mutant (*ga2ox1 ga2ox2 ga2ox3 ga2ox4 ga2ox6 ga2ox7 ga2ox8*; hereafter referred to as *ga2oxhept*). Analysis of nlsGPS1 emission ratios in this line indicated increased GA levels in comparison to Col-0 with more GA being present in the apical hook (Fig. 4D and E; Supplementary Fig. S9). A lesser apical–basal gradient remains, likely because of patterned expression of *GA20ox1*. As with induced *GA20ox1*, the increased level of GA does not affect the pattern of cell elongation of smaller cells nearer the meristem (Fig. 4F), confirming that the correlation of GA levels to cell length observed in the dark-grown hypocotyl is not alone causative for growth patterning.

### Biosynthesis is key for GA patterning in *cop1* mutant

We have demonstrated that GA biosynthesis plays a key role in the formation of the apical–basal gradient with *GA20ox1* and *GA3ox1* being key targets of the COP1 pathway. In order to confirm that the short hypocotyl observed in *cop1-4* is due to an impairment of the biosynthetic pathway and low GA, we created *cop1-4* pUBQ-XVE-AtGA20ox1-P2A-AtGA3ox1 to permit simultaneous β-estradiol induction of ubiquitous *AtGA20ox1* and *AtGA3ox1* in the *cop1-4* background. Hypocotyl length increased with simultaneous β-estradiol induction of ubiquitous *GA20ox1* and *GA3ox1* expression to a similar level as *cop1-4* grown on 1 *µ*m GA_4_ (Supplementary Fig. S10A and B). The increased GA levels and minimal gradient observed in induced *cop1-4* pUBQ-XVE-AtGA20ox1-P2A-AtGA3ox1 confirms that COP1 influencing, likely indirectly, *GA20ox1* and *GA3ox1* expression patterns directs cellular GA distribution (Fig. 4G and H; Supplementary Figs. S11 and S12). Notably, a pattern of increasing cell length was observed in the *cop1-4* background with or without β-estradiol induction of pUBQ-XVE-AtGA20ox1-P2A-AtGA3ox1 and in both cases, cell length was not correlated with cellular GA levels (Fig. 4G and H; Supplementary Figs. S11 and S12). Nonetheless, the broad overaccumulation of GA in the induced state did result in a broad increase in cell length (Fig. 4H and I).

### Next-generation Gibberellin Perception Sensor 2 is reversible and orthogonalized

**Figure 5.**
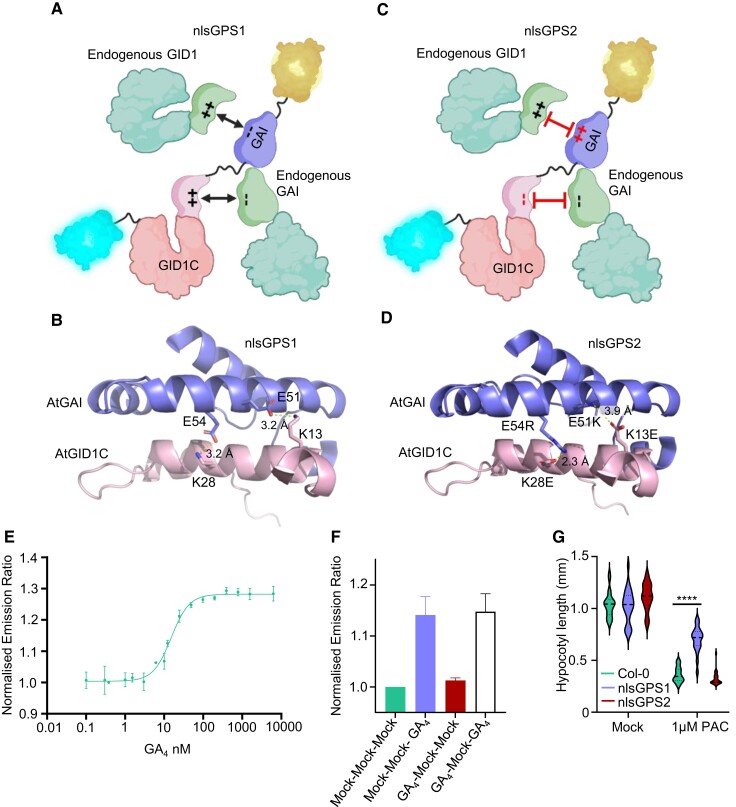
Engineering the orthogonalized and more reversible GPS2 biosensor. **A)** Graphic of interaction of charged amino acids within the sensory domain of GPS1 with endogenous components (BioRender.com). **B)** AlphaFold-predicted structure of the GPS1 interface between GAI and GID1C illustrates the presence of charged residues, E51 and E54 (GAI), and K13 and K28 (GID1C), as well as the presence of 2 predicted electrostatic interactions. **C)** Graphic of the design strategy for GPS2, involving the exchange of 2 pairs of charged residues between GID1C and DELLA in the sensory domain (BioRender.com). **D)** AlphaFold-predicted structure of the GPS2 maintains the electrostatic interactions between the charged residues after undergoing charge exchange mutations (GAI E51K and E54R, GID1C K13E and K28E). **E)** Titration of GPS2 purified protein in response to GA_4_. Changes in the ratio were calculated for GPS2 purified protein with varying GA_4_ concentrations. Error bars represent the variation observed across 5 biological replicates. The dissociation constant (Kd) was calculated as the mean of all biological replicates (*n* = 5). **F)** In vitro reversibility on purified GPS2 protein. Ratio changes were recorded for 2 groups, one with GA pretreatment (GA_4_) and one without (mock). In both groups, the protein was washed through a desalting column twice before GA_4_ treatment (*n* = 2). **G)** Violin plot of hypocotyl lengths of nlsGPS1 and nlsGPS2 lines was compared with wildtype Col-0 with and without paclobutrazol (1 *µ*m PAC) treatment. Student's *t*-test Col-0 to GPS1 and GPS2, ****P-value < 0.0001.

Although GPS1 has high affinity to bioactive GA and reveals cellular distribution and regulation of GA, slow reversibility precluded use for investigation of GA depletion dynamics (Rizza et al. 2017). Both properties are potentially related to the mechanism of GPS1 biosensing involving GA binding the Arabidopsis GA INSENSITIVE DWARF 1C (AtGID1C) moiety which then interacts with the truncated DELLA moiety from Arabidopsis GIBBERELLIC ACID INSENSITIVE AtGAI resulting in a closed biosensor with slow off-rate (Fig. 5A). Another limitation of GPS1 was that the biosensor was not fully orthogonal to endogenous signaling. Stable transgenic lines expressing nlsGPS1 were hyposensitive to GA biosynthesis inhibitor paclobutrazol (PAC) (Rizza et al. 2017), indicating interaction between GPS1 and endogenous GA signaling, likely the AtGID1C moiety with endogenous DELLA proteins (Fig. 5A).

As FRET biosensor re-engineering can deliver improved orthogonality variants (e.g. Rowe et al. 2023), we aimed to reduce GPS1 interference with and by endogenous signaling via re-engineering the GID1–DELLA interaction interface. Analysis of the high-resolution structure of the AtGID1A–AtGAI complex (Shimada et al. 2008) revealed 2 potential interfacial electrostatic interactions that were conserved in GPS1 as AtGID1C Lysine 13 (K13) with AtGAI Glutamate 54 (E54) and AtGID1C Lysine (K28) with AtGAI Glutamate 51 (E51) (Fig. 5B). Introducing charge exchange mutations in the AtGAI moiety (E51K and E54R), which would disrupt the inter-domain interaction, was successfully used to engineer the GPS1-NR negative control sensor (Rizza et al. 2017). We introduced 4 charge exchange mutations K13E and K28E (AtGID1C) and E51K and E54R (AtGAI) into GPS1 to restore the original 2 electrostatic interactions while abrogating interactions between GPS1 and endogenous GID1s and DELLAs (Fig. 5C and D).

In vitro analysis of GPS2 purified from yeast revealed successful restoration of GA emission ratio response and maintenance of a high affinity for bioactive GA_4_ (Fig. 5E). In vitro reversibility testing using buffer-exchange chromatography revealed that this quadruple charge exchange mutant detected a second GA treatment following a first, unlike GPS1 (Fig. 5F). Thus, this biosensor also had improved reversibility, likely due to increased off-rate of the GID1–DELLA interaction resulting from inversion of the interfacial hydrostatic interactions. The charge exchange mutations also appear to have successfully reduced or eliminated interaction with endogenous GID1s and DELLAs as transgenic Arabidopsis lines constitutively expressing a nuclear localized variant of this biosensor were found to have lost the PAC hyposensitivity phenotypes of nlsGPS1 (Fig. 5G). We thus consider the variant to be second generation GIBBERELLIN PERCEPTION SENSOR 2 (GPS2) with improved reversibility and orthogonality.

### Depletion of hypocotyl GA is detected with nlsGPS2 biosensors

**Figure 6.**
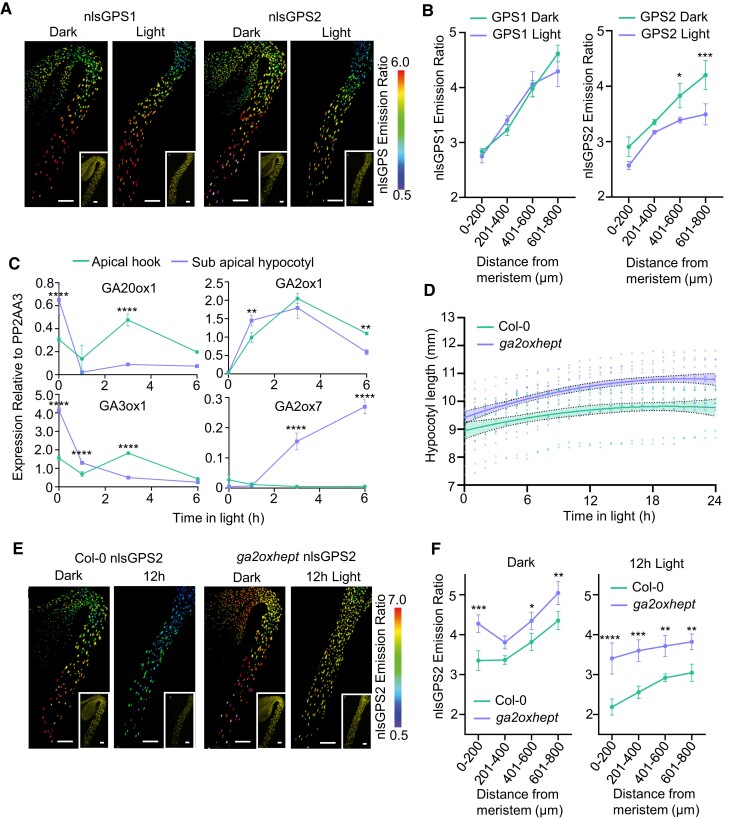
Depletion of hypocotyl GA can be better detected with nlsGPS2 and limited GA depletion in a *ga2oxhept* mutant causes hypocotyl hyperelongation during photomorphogenesis. **A)** Emission ratios of nlsGPS1 and nlsGPS2 in 3 d dark-grown hypocotyls (6 h dark control) and hypocotyls transferred to light for 6 h. Representative images of emission ratios and YFP fluorescence (inset) are shown (additional images and color vision deficiency compatible LUT are shown in Supplementary Fig. S10). Scale bar = 100 *µ*m. **B)** Mean of mean nuclear emission ratios from 3 hypocotyls, binning by distance from the shoot apical meristem of 3 d dark-grown hypocotyls of nlsGPS1 and nlsGPS2 transferred to light for 6 h or maintained in darkness. Error bars = SD. Two-way ANOVA with a Šídák's multiple comparisons post hoc test (*P-value < 0.05, ***P-value < 0.001). **C)** Relative expression changes of selected GA metabolic enzymes in the hook and sub-apical hook of Col-0 during de-etiolation. Dissections were carried out in the dark and at 1, 3, and 6 h light exposure. Data represent mean and standard deviation of 3 technical repeats from 1 biological repeat containing (30) dissected hypocotyls per samples. Experiment was carried out 2 independent times with similar results (second replicate shown in Supplementary Fig. S14C). Two-way ANOVA with a Šídák's multiple comparisons post hoc test (**P-value < 0.01, ****P-value < 0.0001). **D)** Length of 3-d-old dark-grown hypocotyls after lights turned on of Col-0 and *ga2oxhept* mutant. Individual data points plotted with a nonlinear fit of data 2nd order polynomial and 95% confidence interval *r*^2^ = 0.1 and 0.3, respectively. Col-0 *n* = 8, *ga2oxhept n* = 11. Experiment was completed multiple times with similar results. **E)** Emission ratios of Col-0 nlsGPS2 and *ga2oxhept* GPS2 in 3 d dark-grown hypocotyls (12 h dark control) and hypocotyls transferred to light for 12 h. Representative images of emission ratios and YFP fluorescence (inset) are shown (additional images and color vision deficiency compatible LUT are shown in Supplementary Fig. S15). Scale bar = 100 *µ*m. **F)** Mean of mean nuclear emission ratios from 3 hypocotyls, binning by distance from the shoot apical meristem of 3 d dark-grown hypocotyls of Col-0 nlsGPS2 and *ga2oxhept* GPS2 transferred to light for 12 h or maintained in darkness. Significant difference by 2-way ANOVA with a Šídák's multiple comparisons test (Col-0 GPS2 *n* = 3 and *ga2oxhept* GPS2 *n* = 5 biologically independent hypocotyls). *P-value < 0.05, **P-value < 0.01, ***P-value < 0.001, ****P-value < 0.0001.

Previous studies have demonstrated that when a hypocotyl undergoes de-etiolation, there is an increased expression of GA catabolism genes and a decreased expression of some GA biosynthesis genes which correlates to a drop in bioactive GA (Reid et al. 2002; Zhao et al. 2007; Weller et al. 2009). However, there is also a transient increase of some GA biosynthesis genes which could indicate localized GA increase e.g. in the apical hook. How these expression dynamics affect GA dynamics and whether this is relevant to the de-etiolation response were not known. To determine whether the next-generation biosensor could be used to image this decrease of GA, we examined how nlsGPS1 and nlsGPS2 respond during de-etiolation. We imaged the nonreversible Col-0 nlsGPS1 and the reversible Col-0 nlsGPS2 6 h after transfer to the light in comparison with hypocotyls that had been kept in the dark. Col-0 nlsGPS1 did not show a reduction in GA levels, which is contrary to published studies but expected due to the low reversibility the biosensor (Fig. 6A and B; Supplementary Fig. S13). On the other hand, in Col-0 nlsGPS2, we observed a reduction in emission ratios along the length of the hypocotyl with the greatest reduction occurring in cells that had high GA levels in the dark (>600 *µ*m from the meristem) (Fig. 6A and B; Supplementary Fig. S13).

### Limited GA depletion in a *ga2ox* heptuple mutant causes hypocotyl hyperelongation during photomorphogenesis

During the dark to light transition, the distribution of GA in the hypocotyl undergoes reprogramming which we have been able to visualize using nlsGPS2. We observed a greater decrease in the sub-apical hypocotyl than in the apical hook indicating a potential difference in the response to light in these sections of the hypocotyl. To determine potential mechanisms of these differential regulations, we examined the transcripts of GA metabolic genes in whole hypocotyls in the dark and 4 h post-transfer to light and selected metabolic genes in the sub-apical hypocotyl and the apical hook in the dark and 1, 3, and 6 h post-transfer to light.

Both *GA20ox1* and *GA3ox1* transcripts were strongly downregulated in whole hypocotyls at 4 h post-transfer to light (Supplementary Fig. S14A). In dissected hypocotyls, we observed a rapid decline of expression of *GA20ox1* and *GA3ox1* after 1 h in both the apical hook and the sub-apical hypocotyl followed by an increase limited to the apical hook at 3 h (Fig. 6C). The latter result, along with an increase in *GA3ox2* expression in whole hypocotyls after 4 h (Supplementary Fig. S14A), could be an indication of a transient or internally localized GA increase in expanding cells during apical hook opening that was missed in our 6 and 12 h analyses of epidermal and cortical cells using nlsGPS2 (Fig. 6A, B, E, and F). Several *GA2ox* catabolism genes were upregulated in whole hypocotyls in response to 4 h light (Supplementary Fig. S14B). During the dark to light time course, *GA2ox1* expression increased simultaneously in the sub-apical hypocotyl and the apical hook until 3 h (Fig. 6C). In contrast, *GA2ox7* expression was strongly increased but this was limited to the sub-apical hypocotyl region (Fig. 6C). Taken together, differential spatiotemporal expression of *GA20ox1*, *GA3ox1*, and *GA2ox7* could explain the differential extent of GA depletion along the growth axis observed with nlsGPS2 (Fig. 6).

Previous studies have demonstrated that hypocotyl growth arrest during de-etiolation is dependent on GA levels (Folta et al. 2003). We have shown local and temporally dynamic enzyme expression and GA levels in de-etiolating hypocotyls. To establish the importance of reprogramming of GA levels, we observed the growth of the *ga2oxhept* mutant during de-etiolation. The growth rate of Col-0 and *ga2oxhept* mutant diverges at 6 h after transfer to light with Col-0 growth slowing earlier than *ga2oxhept* (Fig. 6D). After 12 h in the light, the Col-0 hypocotyl did not elongate whereas the hypocotyl of the *ga2oxhept* mutant continued to elongate. To investigate the GA levels in the *ga2oxhept* mutant during de-etiolation, we created a *ga2oxhept* nlsGPS2 biosensor line. After 12 h in the light, this line had elevated levels of GA compared to Col-0 nlsGPS2 (Fig. 6E and 6F; Supplementary Fig. S15). Taken together, these results indicate that upon transfer to light, reduced biosynthesis and increased catabolism in the hypocotyl lead to decreased GA levels and this depletion is quantitatively important for slowing elongation.

## Discussion

Plant developmental plasticity emerges in part via integration of environmental signals into hormone dynamics, with distinct outcomes based on developmental context. For both embryo protrusion from seed coats in germination and seedling protrusion through soil in skotomorphogenesis, GA accumulation coordinates endogenous signals with environmental cues, particularly light and darkness, to appropriately direct organ growth. In Arabidopsis seed germination, PIF1 antagonizes GA biosynthesis and promotes its catabolism (Oh et al. 2004; Gabriele et al. 2010), thus linking light with GA accumulation. Using the direct GA biosensor nlsGPS1, we previously uncovered a GA accumulation gradient in skotomorphogenic hypocotyl cells that was absent in photomorphogenesis (Rizza et al. 2017). It was known that light antagonizes GA in this developmental context, but the complexity of light signaling and GA metabolism in Arabidopsis provided multiple potential points of regulation for cellular GA dynamics.

Here, we investigated the biochemical basis of this hypocotyl GA gradient and discovered that COP1 signaling acting, presumably indirectly, on the patterned expression of the gene encoding the GA20ox1 biosynthetic enzyme is an important regulatory module in skotomorphogenesis. We also show, using a *ga2oxhept* mutant, that GA catabolism contributes to articulating the gradient in the dark. Although we previously showed the *pifq* mutant did have lower GA levels in the dark, we now demonstrate that PIFs were dispensable for the GA gradient formation and were instead key for maintenance of skotomorphogenesis. Brassinosteroid signaling has been shown to both promote and repress GA accumulation (Unterholzner et al. 2015; Albertos et al. 2022), but providing epibrassinolide or BRZ inhibitor did not abolish the GA gradient. As PIF and BR patterning are unlikely to explain *GA20ox1* expression in the sub-apical hypocotyl and HY5 activity is limited in the dark, determining the responsible regulators for which COP1 signaling is permissive will be an interesting topic for a future study.

The discovery of a GA gradient correlated with cell length in etiolated hypocotyls was not evidence of GA levels acting in a simple dose-response relationship with elongation. Rather, overaccumulation of GA in the *ga2oxhept* mutant and after induction of *GA20ox1* expression did not alter cell elongation patterning and thus we can conclude that GA accumulation is not alone sufficient to set cell length in the dark. A similar dynamic was determined in Arabidopsis root elongation in the light (Rizza et al. 2017). Nonetheless, GA is required for sub-apical hypocotyl cell elongation and a spatially resolved lowering of GA levels will be required to fully reveal the quantitative relationship between GA and the cell elongation gradient. A model in which local GA concentrations cooperate with levels of other growth factors (e.g. PIFs and brassinosteroids) to set cell length would have distinct implications for morphogenesis control than, for example, one in which a low threshold of GA potentiates growth (i.e. via DELLA degradation) with other factors setting cell length. For example, in a cooperative model, a range of GA concentrations would be meaningful and provide robustness for cell length patterning while in a thresholded model, GA would provide a gating function for growth.

While relative depletion of GA from the apical hook was not important in skotomorphogenesis, we showed that catabolism of GA was quantitatively important in the early stages of photomorphogenesis. The engineering of GPS2 to have improved orthogonality (i.e. reduced or eliminated interaction with endogenous components) and reversibility over GPS1 enabled us to better track GA depletion during de-etiolation and *ga2oxhept* mutant hypocotyls over elongated after illumination. As either local or temporal GA control is meaningful in many plastic traits, for example axial shoot fate switching in grapevine (*Vitis vinifera*) and woodland strawberry (*Fragaria vesca*) and inflorescence architecture in rice (*Oryza sativa*) (Wu et al. 2016; Tenreira et al. 2017; Zheng et al. 2018), our study serves as a model for using GPS2 in vivo to quantify the relationship between environmentally sensitive cellular GA dynamics and developmental plasticity. Indeed, the nlsGPS2 biosensor was transiently introduced and shown to be functional in several monocot crop species (Dao et al. 2023) and stably introduced into *Medicago truncatula* to clarify where and when root cells accumulate GA during rhizobium induced nodulation, which had previously been shown to be both promoted and inhibited by GA (Drapek et al. 2023).

## Materials and methods

### Plant materials and growth conditions

WT, mutant, and transgenic lines seed in this study were Arabidopsis (*A. thaliana*) ecotype Columbia (Col-0) with the exception of *phyA phyB cry1 cry2* in the Landsberg (Ler) background. Seeds were chlorine gas-sterilized and plated on ½ Murashige and Skoog (MS) basal medium (Duchefa Biochemie) with 0.025% (w/v) MES (Sigma, pH 5.7 and 1.2% agar (Duchefa Biochemie). Seeds were stratified in the dark at 4 °C for 3 d. Light-grown seedlings were placed in a growth chamber with long-day growth conditions (120 *μ*mol m^−2^ s^−1^ white light, 22 °C for 16 h; 0 *μ*mol m^−2^ s^−1^, 18 °C for 8 h). For dark-grown seedlings, plates were transferred to 22 °C light for 4 h before being wrapped in 2 layers of foil to simulate constant darkness and returned to the growth chamber with long-day growth conditions (16 h/8 h temperature cycling 22 °C/18 °C).

For β-estradiol induction of pUBQ-XVE-AtGA20ox1, pUBQ-XVE-AtGA20ox1-P2A-AtGA3ox1, and pUBQ-XVE-AtGA3ox1, lines (Rizza et al. 2021) in the dark seeds were plated on a fine mesh (100 mu nylon, 38% open area, Normesh), transfer was carried out under green light to ½ MS solid medium supplemented with 2.5 *µ*m 17-β-estradiol for the time indicated in the text. Mock induction with DMSO was used as a control. For hormone treated seedlings, lines were plated on a fine mesh, 24 h after transfer to the growth chamber, the mesh was transferred to plates containing the appropriate chemical.

### Generation of transgenic Arabidopsis lines

The following nlsGPS1 and nlsGPS2 Arabidopsis lines were generated during this work using the Agrobacterium floral dip method and transformants were selected on ½ MS agar plates containing kanamycin Col-0 nlsGPS2, *cop1-4* nlsGPS1, *hy5-215* nlsGPS1, *ga2oxhept* GPS1, *ga2oxhept* GPS2, and *phyA phyB cry1 cry2* nlsGPS1. To generate *ga2oxhept* homozygous mutant containing null alleles of 7 *GA2ox* genes, *ga2ox7-2* (SALK_055721) and *ga2ox8* (SALKseq_040686.2) were crossed to generate *ga2ox7 ga2ox8*. This was then crossed to *ga2oxq* (Rieu et al. 2008), subsequent generations were backcrossed to *ga2oxq* and until the homozygous line was generated. To generate *cop1-4 hy5-215* nlsGPS1, *cop1-4* nlsGPS1 was crossed to *cop1-4 hy5-215*, the F1 selfed, and the homozygous line identified in the F2. Generation of *cop1-4* pUBQ10-XVE-GA20ox1-P2A-GA3ox1 nlsGPS1 was achieved by crossing *cop1-4* to pUBQ10-XVE-GA20ox1-P2A-GA3ox1 nlsGPS1. Homozygous lines were confirmed by using PCR-based genotyping using allele-specific primers (Supplementary Table S1); those containing nlsGPS1 were screened at seedling stage for fluorescence, and those containing inducible lines were screened on hygromycin.

### Engineering and characterization of GPS2 biosensor

Site direct mutagenesis of GPS1 (plasmids pDR-FLIP43 GPS1 and p16-FLIP43 nlsGPS1 (Rizza et al. 2017) was achieved using QuikChange site-directed mutagenesis kit and Phusion high-fidelity DNA polymerase (NEB) following manufacturer protocol resulting in plasmids pDR-FLIP43 GPS2 (for yeast expression) and p16-FLIP43 nlsGPS2 (for plant expression). Expression and fluorescence characterization of biosensors in protease-deficient yeast were performed as described previously (Jones et al. 2014). *Saccharomyces cerevisiae* strain BJ5465 (ATCC 208289 *MATa ura3-52 trp1 leu2-*Δ*1 his3-*Δ*200 pep4:HIS3 prb1-*Δ*1.6 R can1 GAL*) was transformed with plasmid pDR-FLIP43 GPS2 and selected on synthetic complete (SC) medium with 0.8% agar supplemented with 240 mg/l leucine and 20 mg/l tryptophan (SC agar +Leu, +Trp) for complementation of uracil auxotrophy by the URA3 marker. Transformed yeast was grown in 50 ml SC medium-Ura in 250 ml flasks for 2 nights. Following, centrifugation, 500 to 700 *µ*l chilled silicon bead slurry (MOPs buffer, 0.1% Triton X-100, and 50% [v/v] Zirconia/Silica beads [0.5 mm; Biospec]) was added to each tube containing the cell pellets. The tubes were vortexed for 15 to 20 min at 4 °C. The cell lysate was centrifuged at 10,000 × *g* for 10 min at 4 °C. Yeast lysates were diluted 1:2 in 20 mm Tris-HCl, 5 mm imidazole, pH 7.4 and then bound to poly-prep chromatography columns containing His-bind resin (Novagen). The yeast lysate mixture was loaded in the columns, and a 30-min rolling at 4 °C was carried out to allow bonding. Columns were washed twice with 50 mm MOPs, 10 mm imidazole, pH 7.4 and eluted in 50 mm MOPS, 150 mm imidazole, pH 7.4. The eluate was diluted with 50 mm MOPS, pH 7.4 in clear-bottom 96-well plates (Greiner) with GA_4_ concentrations as indicated (Sigma) and analyzed with a fluorometer (SpectraMax, Molecular Biosciences). Fluorescence readings were acquired for the acceptor FP emission wavelength (acceptor emission: Am) when samples were excited with the excitation wavelength for acceptor FP (acceptor excitation: Ax) as the control of sensor expression. An emission scan, including donor and acceptor emission spectra, was acquired when exciting with donor fluorophore excitation wavelength (donor excitation: Dx). The emission scan range was set between 470 and 550 nm with a step size of 5 nm and excitation at 428 nm. For all imaging, the bandwidth was set to 12 nm, the number of flashes 10, and the integration time 40 *µ*s. BJ5465 yeast containing an empty vector was used as a negative control for background subtraction. GraphPad was used to analyze affinity. Technical replicates were averaged to determine the mean emission ratio for each sample with or without ligand. The intensity of emission of a donor over the intensity of emission of FRET (DxAM/DxDm) was calculated as the emission ratio. Titration curves were analyzed using GraphPad software.

### Reversibility determination

Zeba Spin Desalting Columns, 0.5 ml, 40K MWCO (Thermo Scientific) were used to perform in vitro reversibility binding test. Purified sensors were pretreated with 0 *µ*m GA_4_ and 0.1 *µ*m GA_4_. After two 20-min buffer-exchange steps with 50 mm MOPS pH 7.4 using Zeba spin columns, the eluted GPS2 in 50 mm MOPS pH 7.4 was treated with 0 *µ*m GA_4_ and 0.1 *µ*m GA_4_. Fluorescence emission ratios were analyzed as above in clear-bottom 96-well plate with a SpectraMax fluorometer.

### Phenotypic characterization

For hypocotyl length assays, plants were grown vertically as described in growth conditions. Plates were imaged after 3 d for dark-grown hypocotyls and hypocotyls lengths were measured using Fiji software. At least 3 independent experiments were performed and the sample size is indicated in the figure legends.

### Infrared imaging and analysis

For imaging dark-grown hypocotyls and de-etiolation, a custom IR imaging setup was used. Images were acquired at 1 h intervals. For hook angle experiments, seeds were plated horizontally to reduce gravity effects on hook formation (Zhu et al. 2019; Du et al. 2022). At 72 h after start of germination light pulse, the chamber light was turned on. Hypocotyl lengths and the angles between the cotyledons and the hypocotyl were measured using the Fiji software. At least 3 independent experiments were performed and the sample size is indicated in the figure legends.

### RNA extraction and expression analysis

Seedlings grown for hypocotyl RNA extraction were grown on Normesh in either long day or dark conditions for 3 d. Dark to light transition samples were grown in the dark for 3 d and transferred to light for 4 h before harvest. The hypocotyls were then collected for RNA extraction. Total RNA was extracted using RNeasy Plant Mini Kit (Qiagen). After DNase digest (TURBO DNase, Invitrogen), 1 *µ*g total RNA was used for cDNA synthesis (SuperScript VILO cDNA Synthesis Kit, Invitrogen). The expression of genes was tested by RT-qPCR (LightCycler 480 SYBR Green I, Roche).

Seedlings grown for RNA extraction from the hook and the hypocotyl were grown on a fine strip of Normesh such that the hypocotyl extended above the mesh onto the media. Dissection was carried out following a procedure adapted from a grafting protocol (Melnyk 2017). Total RNA was extracted using RNAqueous-Micro Total RNA Isolation Kit (Ambion). After DNase digest (TURBO DNase, Invitrogen), 0.5 *µ*g total RNA was used for cDNA synthesis (SuperScript VILO cDNA Synthesis Kit, Invitrogen). The expression of genes was tested by RT-qPCR (LightCycler 480 SYBR Green I, Roche). Primers were listed in Supplementary Table S1. PROTEIN PHOSPHATASE 2A SUBUNIT A3 (PP2AA3; Atg1g13320) was used as the internal control. The analysis of relative gene expression data was performed using the 2(−ΔΔC[T]) method. Replication is described in each relevant figure legend.

### GUS staining

Etiolated 3-d-old seedlings were incubated for 4 h at 37 °C in the GUS buffer (200 mm NaH_2_PO_4_, pH 7.0, 0.5 m EDTA pH 8.0, 0.1% Triton-X, 20 mm potassium ferricyanide, and 20 mm potassium ferrocyanide containing 20 mm 5-bromo-4-cholro-3-indolyl glucuronide). To stop the GUS reaction, the reaction mix was replaced with an ethanol concentration series (30 min minimum) at 30%, 50%, 70%, and 100%. Seedlings were imaged with a Keyence microscope.

### Confocal imaging

Seedlings were mounted in liquid ¼ × MS medium (1/4 × MS salts, 0.025% MES (w/v), pH 5.7), covered with a coverslip and imaged. Confocal images were acquired with a format of 1,024 × 512 pixels and a resolution of 12 bit on an upright Leica SP8FLIMan using a 10× dry objective for dark-grown hypocotyls and a 20× dry 0.70 HC PLAN APO objective for light-grown hypocotyls. To excite Cerulean and Aphrodite, 448 and 514 nm lasers were used, respectively. The 552 nm laser line was used to excite propidium iodide (PI, Sigma-Aldrich). Emission filters were 460 to 500 nm for Cerulean, 525 to 560 nm for Aphrodite, and 590 to 635 nm for PI. Three fluorescence channels were collected for FRET imaging: Cerulean donor excitation and emission or DxDm, Cerulean donor excitation, Aphrodite acceptor emission or DxAm, and Aphrodite acceptor excitation and emission or AxAm. The laser power was set to 3% to excite Cerulean and 2% to excite Aphrodite with detector gain set to 110.

### Imaging processing and analysis

Imaging process and analysis were performed with FRETENATOR plugins (Rowe et al. 2022) Segmentation settings were optimized for each experiment but kept constant within each experiment. The AxAm channel was used for segmentation. For segmentation, Otsu thresholds were used, difference of Gaussian kernel size was determined empirically, and a minimum ROI size was set to 20. Distance from meristem was defined using FRETENATOR ROI labeler.

### Data visualization and statistical analysis

GraphPad was used for plotting and statistical analysis. All statistical tests are described in the figure legends with sample size. Statistical data are provided in Supplementary Data Set 1.

### Accession numbers

Genes studied can be found in the Arabidopsis TAIR database (https://www.arabidopsis.org) under the following accession numbers: *GA20OX1* (AT4G25420), *GA20OX2* (AT5G51810), *GA20OX3* (AT5G07200), *GA3OX1* (AT1G15550), *GA3OX2* (AT1G80340), *PHYA* (AT1G09570), *PHYB* (AT2G18790), *CRY1* (AT4G08920), *CRY2* (AT1G04400), *COP1* (AT2G32950), *HY5* (AT5G11260), *GA2OX1* (AT1G78440), *GA2OX2* (AT1G30040), *GA2OX3* (AT2G34555), *GA2OX4* (AT1G47990), *GA2OX6* (AT1G02400), *GA2OX7* (AT1G50960), and *GA2OX8* (AT4G21200). Lines used in this article can be found in NASC under the following accession numbers: N2110211: *ga20ox1, ga20ox2, ga20ox3* nlsGPS1; N2110213: pUBQ10-XVE-GA20ox1 nlsGPS1; N2110214: pUBQ10-XVE-GA3ox1 nlsGPS1; N2110216: pUBQ10-XVE-GA20ox1-P2A-GA3ox1 nlsGPS1; N2107737: *pifq* nlsGPS1; and N2107734: Col-0 nlsGPS1.

## Supplementary Material

koae198_Supplementary_Data

## Data Availability

Biological data have been deposited in Apollo Open Access Cambridge Data Repository doi: 10.17863/CAM.110541.

## Supplementary data

The following materials are available in the online version of this article.

**Supplementary Figure S1.** Additional images of Col-0 nlsGPS1 and *pifq* nlsGPS1 dark-grown hypocotyls (supports Fig. 1C and D).

**Supplementary Figure S2.** Additional images of Col-0 nlsGPS1 dark-grown hypocotyls treated with EBR and BRZ (supports Fig. 1F and G).

**Supplementary Figure S3.** Additional images of Col-0 nlsGPS1, *cop1-4* nlsGPS1, and *cop1-4 hy5-215* nlsGPS1 dark-grown hypocotyls (supports Fig. 2C and D).

**Supplementary Figure S4.** Additional images of Col-0 nlsGPS1 and *hy5-215* nlsGPS1 dark-grown hypocotyls (supports Fig. 2E and F).

**Supplementary Figure S5.** Hook angles 26 h after germination (supports Fig. 2).

**Supplementary Figure S6.** Relative expression of GA metabolic genes in Col-0, cop1-4, and cop1-4 hy5-215 dark-grown hypocotyls (supports Fig. 2).

**Supplementary Figure S7.** Additional images of GA enzyme induction in Col-0 nlsGPS1 (supports Fig. 3G to I).

**Supplementary Figure S8.** Additional images of Col-0 nlsGPS1 and ga20ox1 ga20ox2 ga20ox3 nlsGPS1 dark-grown hypocotyls (supports Fig. 4B and C).

**Supplementary Figure S9.** Additional images of Col-0 nlsGPS1 and *ga2oxhept* nlsGPS1 dark-grown hypocotyls (supports Fig. 4D and E).

**Supplementary Figure S10.** Hypocotyl length after GA enzyme induction in *cop1-4* nlsGPS1 (supports Fig. 4).

**Supplementary Figure S11.** Additional images of GA enzyme induction in *cop1-4* nlsGPS1 (supports Fig. 4G and H).

**Supplementary Figure S12.** Emission ratio of nlsGPS1 in Col-0 and mock treated and induced *cop1-4* pUBQ-XVE-AtGA20ox1-P2A-AtGA3ox1 nlsGPS1 (supports Fig. 4).

**Supplementary Figure S13.** Additional images of dark to light transition Col-0 nlsGPS1 vs Col-0 nlsGPS2 (supports Fig. 6A and B).

**Supplementary Figure S14.** Gene expression in hypocotyls after transfer to light (supports Fig. 6C).

**Supplementary Figure S15.** Additional images of dark to light transition Col-0 nlsGPS2 vs *ga2oxhept* nlsGPS2 (supports Fig. 6E and F).

**Supplementary Table S1.** Primers used in this study.

**Supplementary Data Set 1.** Statistical data.

## Dive Curated Terms

The following phenotypic, genotypic, and functional terms are of significance to the work described in this paper:

GPS1 Gramene: AT2G34630
GPS1 Araport: AT2G34630

BZR1 Gramene: AT1G75080
BZR1 Araport: AT1G75080

GA3ox2 Gramene: AT1G80340
GA3ox2 Araport: AT1G80340

GIBBERELLIN CHEBI: CHEBI:24250

ga CHEBI: CHEBI:5206

GA20ox1 Gramene: At4g25420
GA20ox1 Araport: At4g25420

GA20ox2 Gramene: AT5G51810
GA20ox2 Araport: AT5G51810

GA2ox6 Gramene: AT1G02400
GA2ox6 Araport: AT1G02400

GA2ox7 Gramene: AT1G50960
GA2ox7 Araport: AT1G50960

GA2OX8 Gramene: AT4G21200
GA2OX8 Araport: AT4G21200

GA3ox1 Gramene: At1g15550
GA3ox1 Araport: At1g15550

HY5 Gramene: AT5G11260
HY5 Araport: AT5G11260

COP1 Gramene: AT2G32950
COP1 Araport: AT2G32950

GAI Gramene: AT1G14920
GAI Araport: AT1G14920

pifq Gramene: pif1 pif3 pif4 pif5
pifq Araport: pif1 pif3 pif4 pif5
