# Supplementary material for: GIBBERELLIN PERCEPTION SENSOR 2 reveals genesis and role of cellular GA dynamics in light-regulated hypocotyl growth
Source: Plant Cell. 2024 Jul 23;36(10):4426–41. doi: 10.1093/plcell/koae198 (PMC11449061; doi:10.1093/plcell/koae198)
Supplement: koae198_Supplementary_Data [file koae198_supplementary_data.zip › TPC2024RA00366D_Supplementary Figures and Table.pdf]

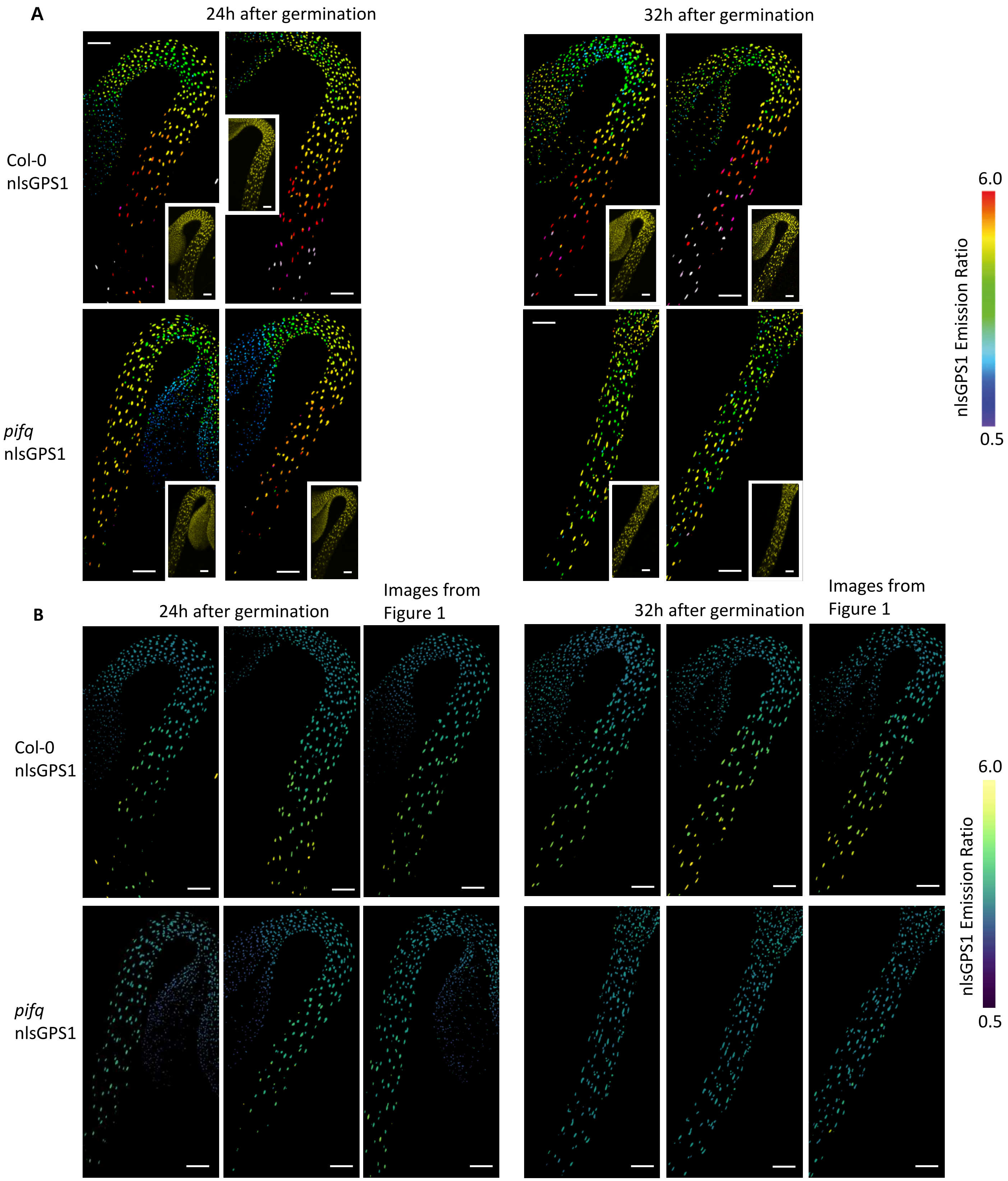

Supplementary Figure S1. Additional images of Col-0 nlsGPS1 and *pifq* nlsGPS1 dark grown hypocotyls. (Supports Figure 1c and d). nlsGPS1 emission ratios of dark grown hypocotyls 24h and 32h after germination. A) 16_colors LUT. Representative images of emission ratios and YFP fluorescence (Inset) are shown. B) Color vision deficiency compatible LUT (Viridis) (Images corresponding to emission ratio images presented in Figure 1c and d). Scale bar = 100µm

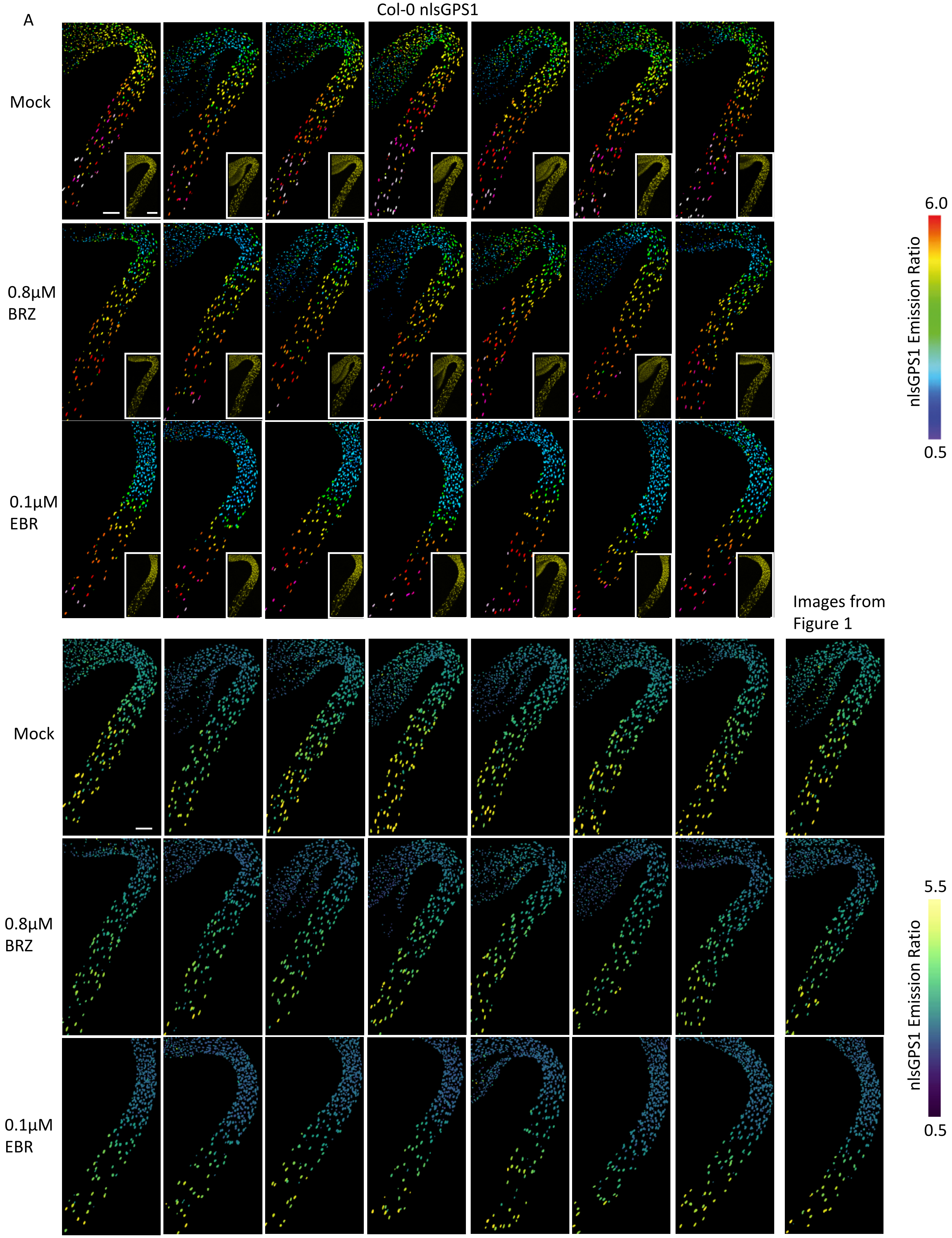

Supplementary Figure S2. Additional images of Col-0 nlsGPS1 dark grown hypocotyls treated with EBR and BRZ. (Supports Figure 1f and g). nlsGPS1 emission ratios of dark grown hypocotyls treated with 0.1µM epi-brassonolide or 0.8µM brassinazole for 24h before imaging with DMSO treatment as mock. A) 16_colors LUT. Representative images of emission ratios and YFP fluorescence (Inset) are shown. B) Color vision deficiency compatible LUT (Viridis) nuclei dilated (size increased) after analysis to allow easier visual discrimination of this LUT. (Images corresponding to emission ratio images presented in Figure 1f and g). Scale bar = 100µm applies to all images

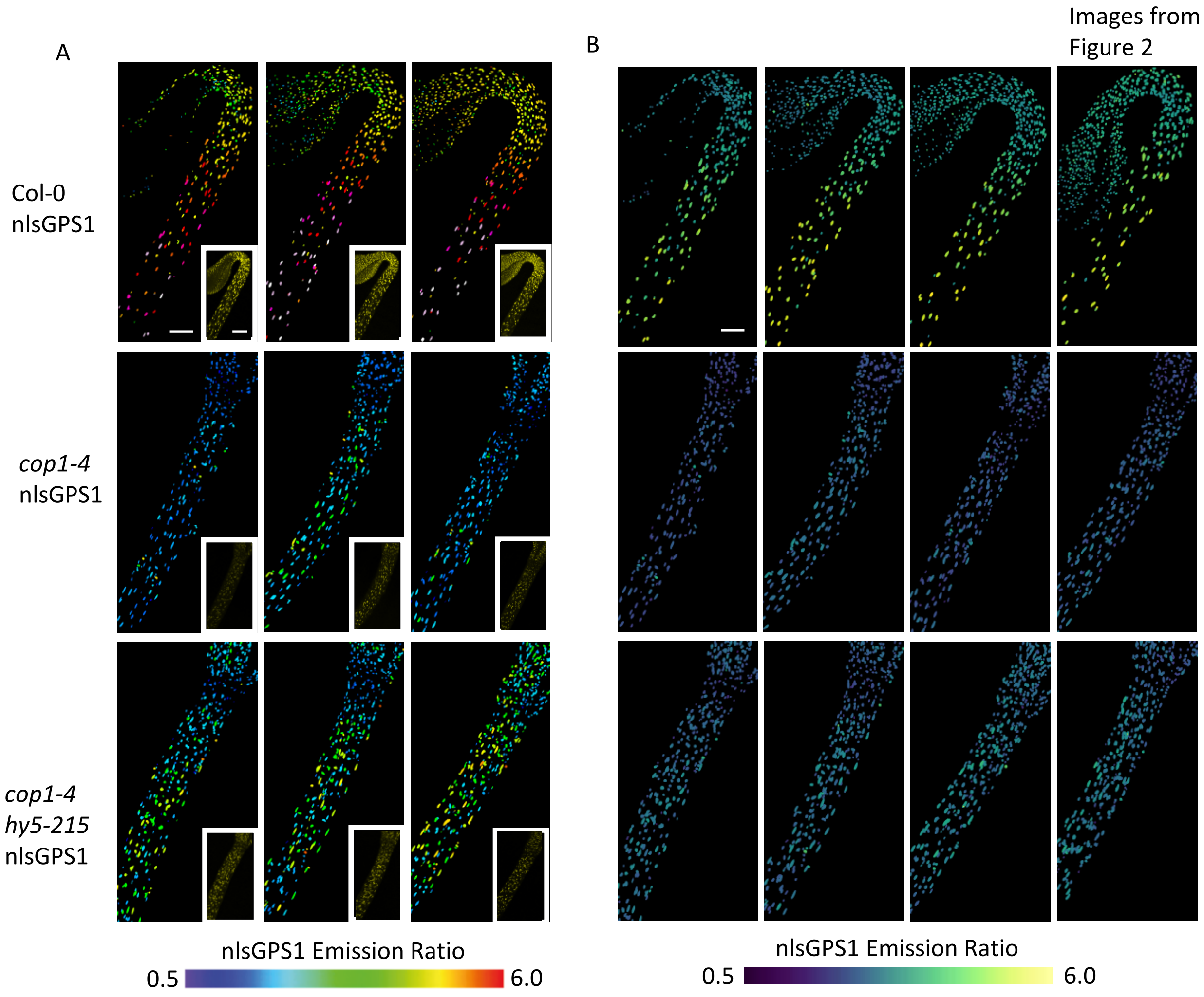

Supplementary Figure S3. Additional images of Col-0 nlsGPS1, *cop1-4* nlsGPS1 and *cop1-4 hy5-215* nlsGPS1 dark grown hypocotyls. (Supports Figure 2c and d). nlsGPS1 emission ratios of 3 day old dark grown hypocotyls. A) 16_colors LUT. Representative images of emission ratios and YFP fluorescence (Inset) are shown. B) Color vision deficiency compatible LUT (Viridis) nuclei dilated (size increased) after analysis to allow easier visual discrimination of this LUT. (Images corresponding to emission ratio images presented in Figure 2c and d). Scale bar = 100µm applies to all images

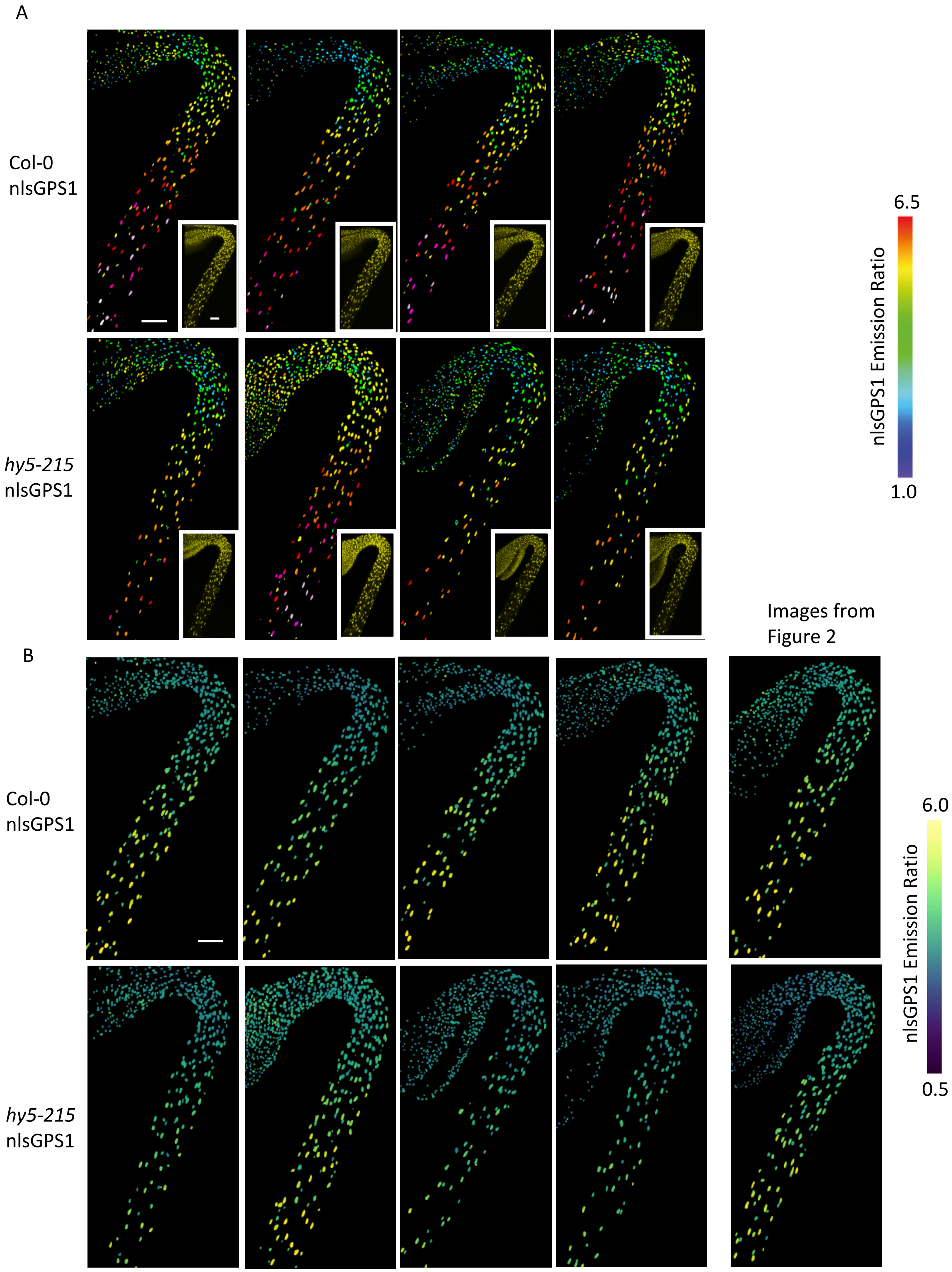

Supplementary Figure S4. Additional images of Col-0 nlsGPS1 and *hy5-215* nlsGPS1 dark grown hypocotyls. (Supports Figure 2e and f). nlsGPS1 emission ratios of 3 day old dark grown hypocotyls. A) 16_colors LUT. Representative images of emission ratios and YFP fluorescence (Inset) are shown. B) Color vision deficiency compatible LUT (Viridis) nuclei dilated (size increased) after analysis to allow easier visual discrimination of this LUT. (Images corresponding to emission ratio images presented in Figure 2e and f). Scale bar = 100µm applies to all images

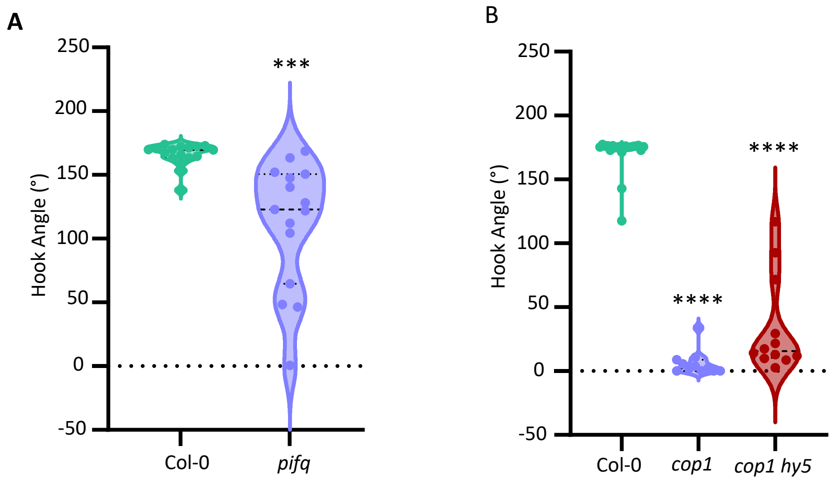

Supplementary Figure S5. Hook angles 26 hours after germination. (Supports Figure 2). A) Hook angles of Col-0 and *pifq* 26 hours after germination (data from Figure 1b). An unpaired t-test was performed to compare hook angle of Col-0 and *pifq*. B) Hook angles of Col-0, *cop1-4* and *cop1-4 hy5-215* 26 hours after germination. A one-way ANOVA was performed to compare the effect of genotype on hook angle. A Dunnet's post hoc test was used for multiple comparisons to Col-0 control ****p-value < 0.0001.

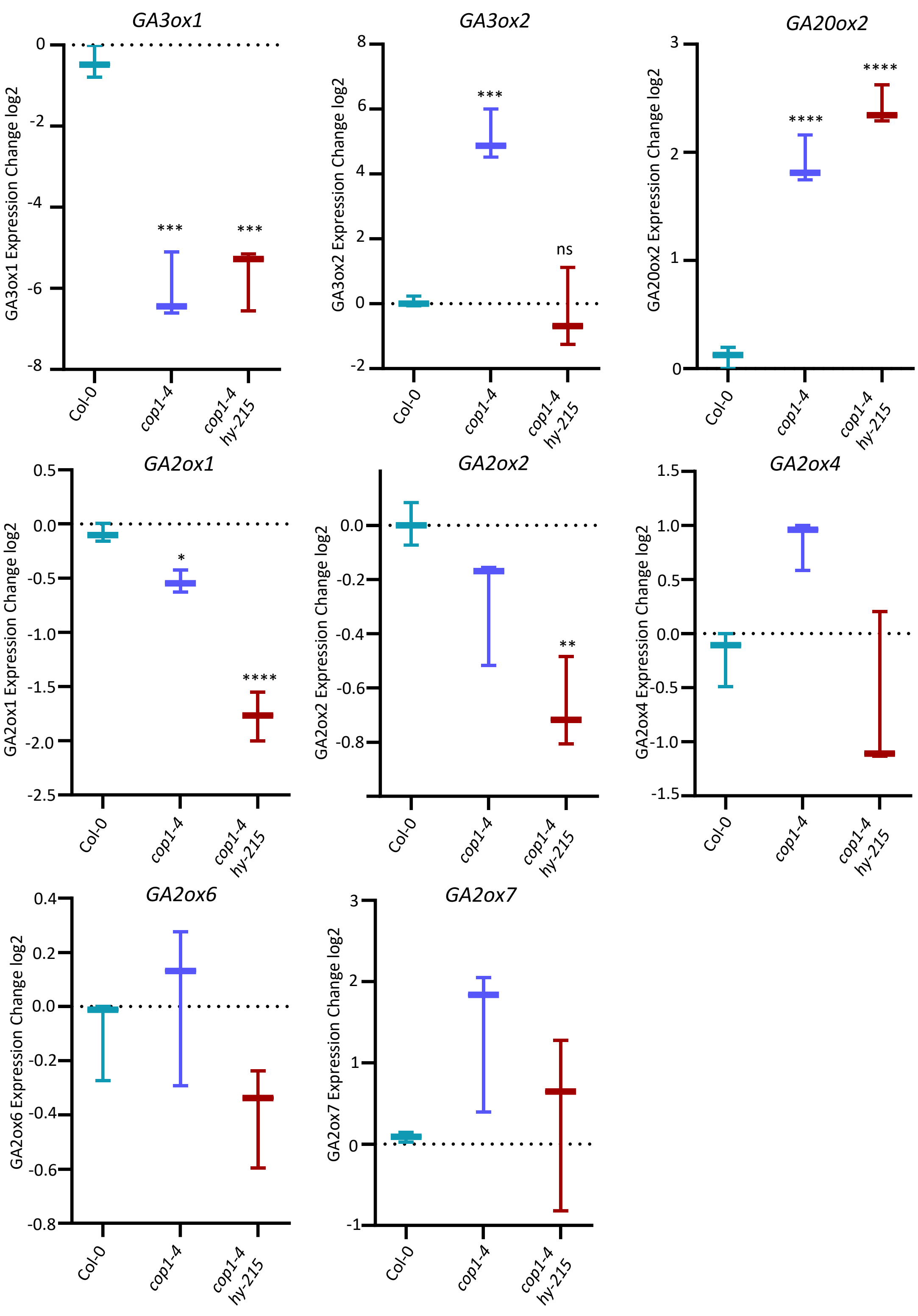

Supplementary Figure S6. Relative expression of GA metabolic genes in Col-0, *cop1-4* and *cop1-4 hy5-215* dark grown hypocotyls. (Supports Figure 2). Log2 fold change from Col-0 for *cop1-4* and *cop1-4 hy5-215* are shown. Reference gene PP2AA3. A one-way ANOVA was performed to compare the effect of genotype on gene expression. A Dunnet's post hoc test was used for multiple comparisons to Col-0 control * p-value <0.05, **p-value < 0.01, ***p-value < 0.001, ****p-value < 0.0001

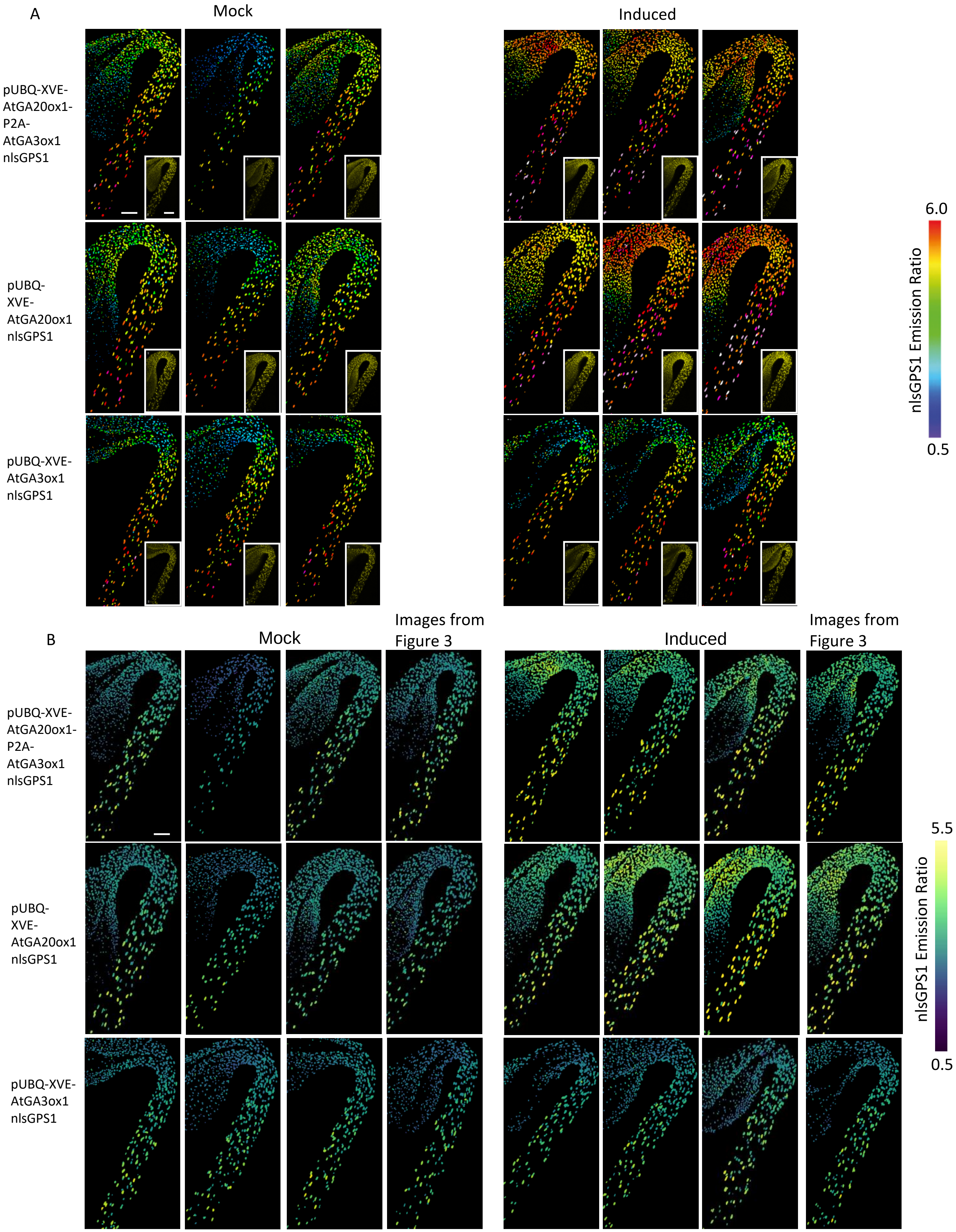

Supplementary Figure S7. Additional images of GA enzyme induction in Col-0 nlsGPS1. (Supports Figure 3g-i). nlsGPS1 emission ratios of 3 day old dark grown hypocotyls β-estradiol inducible GA enzyme transgenic lines 48 h after induction with 2.5 µM 17-β-estradiol (induced) or with 0.1% DMSO mock induction (mock). A) 16_colors LUT. Representative images of emission ratios and YFP fluorescence (Inset) are shown. B) Color vision deficiency compatible LUT (Viridis) nuclei dilated (size increased) after analysis to allow easier visual discrimination of this LUT. (Images corresponding to emission ratio images presented in Figure 3g-i). Scale bar = 100µm applies to all

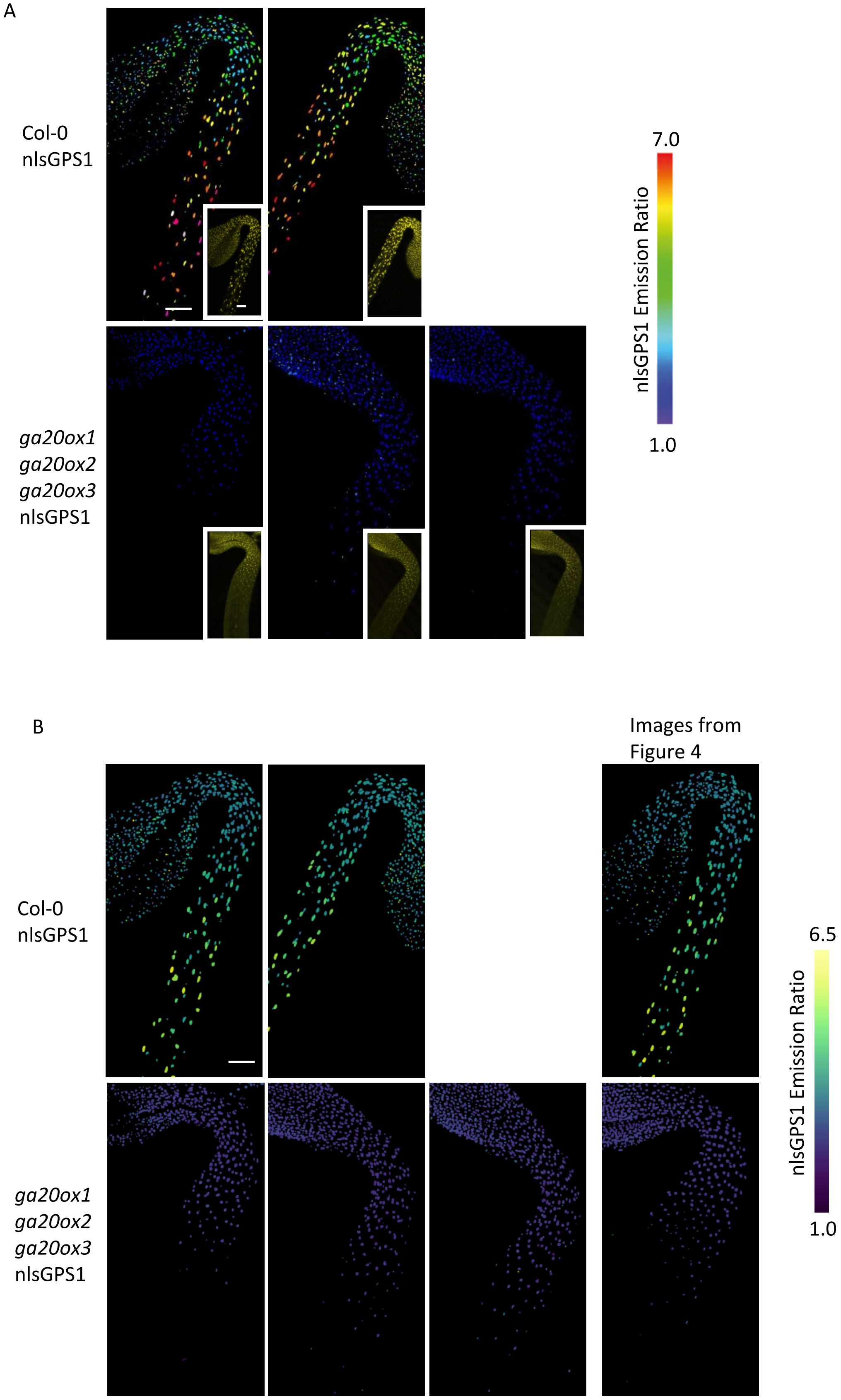

Supplementary Figure S8. Additional images of Col-0 nlsGPS1 and *ga20ox1 ga20ox2 ga20ox3* nlsGPS1 dark grown hypocotyls. (Supports Figure 4b and c). nlsGPS1 emission ratios of 3 day old dark grown hypocotyls. A) 16_colors LUT. Representative images of emission ratios and YFP fluorescence (Inset) are shown. B) Color vision deficiency compatible LUT (Viridis) nuclei dilated (size increased) after analysis to allow easier visual discrimination of this LUT. (Images corresponding to emission ratio images presented in Figure 4b and c). Scale bar = 100µm applies to all images

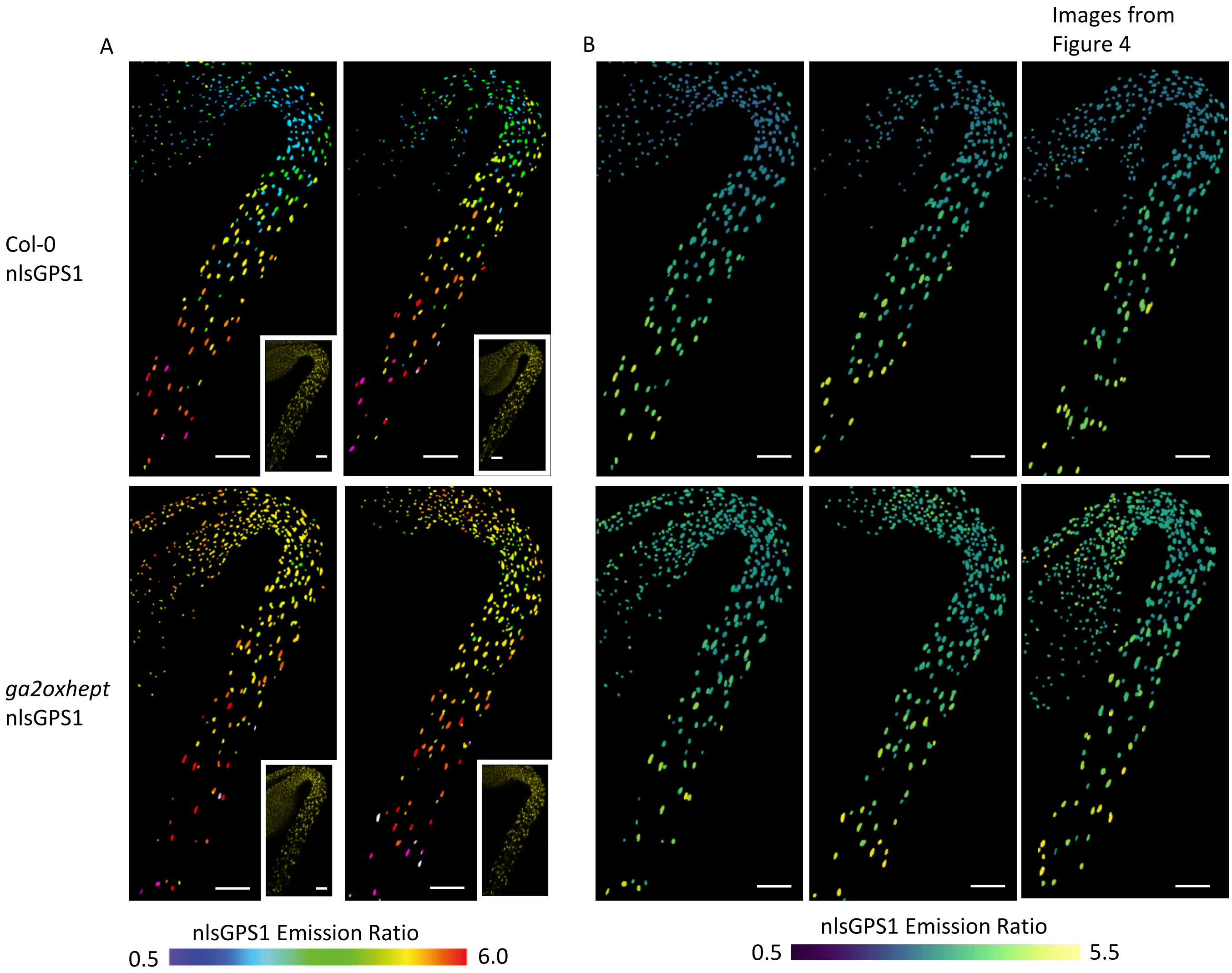

Supplementary Figure S9. Additional images of Col-0 nlsGPS1 and *ga2oxhept* nlsGPS1 dark grown hypocotyls. (Supports Figure 4d and e). nlsGPS1 emission ratios of 3 day old dark grown hypocotyls. A) 16_colors LUT. Representative images of emission ratios and YFP fluorescence (Inset) are shown. B) Color vision deficiency compatible LUT (Viridis) nuclei dilated (size increased) after analysis to allow easier visual discrimination of this LUT. (Images corresponding to emission ratio images presented in Figure 4d and e). Scale bar = 100µm

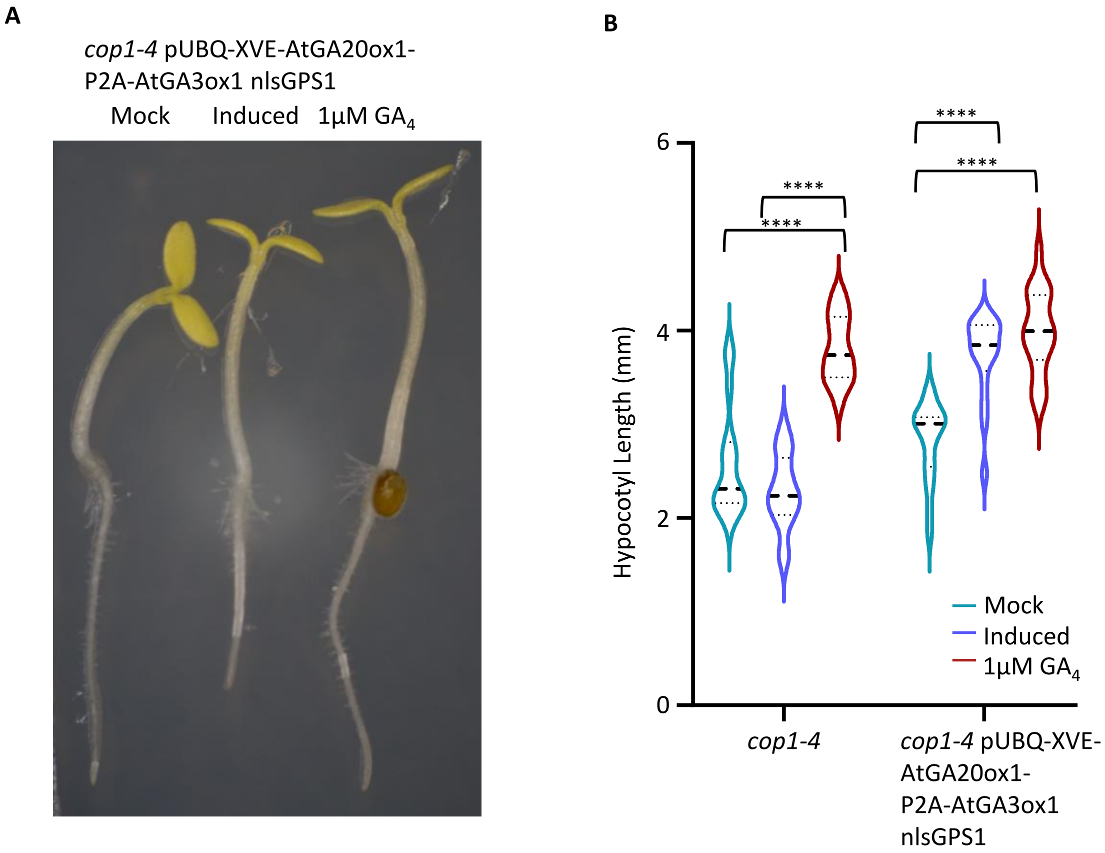

Supplementary Figure S10. Hypocotyl length after GA enzyme induction in *cop1-4* nlsGPS1. (Supports Figure 4). A) Representative images of *cop1-4* pUBQ-XVE-AtGA20ox1-P2A-AtGA3ox1 nlsGPS1 3 day old dark grown hypocotyls 48 h after mock induction with 0.1% DMSO (mock), induction with 2.5 µM 17-β-estradiol or on media containing 1µM GA_4. B) Violin plot of hypocotyl lengths of *cop1-4* and *cop1-4* pUBQ-XVE-AtGA20ox1-P2A-AtGA3ox1 nlsGPS1 3 day old dark grown hypocotyls 48 h after mock induction with 0.1% DMSO (mock), induction with 2.5 µM 17-β-estradiol or on media containing 1µM GA_4. Two-way ANOVA with a Tukey's post hoc test (n> 16 biologically independent hypocotyls for all genotypes and treatments). ****p-value < 0.0001.

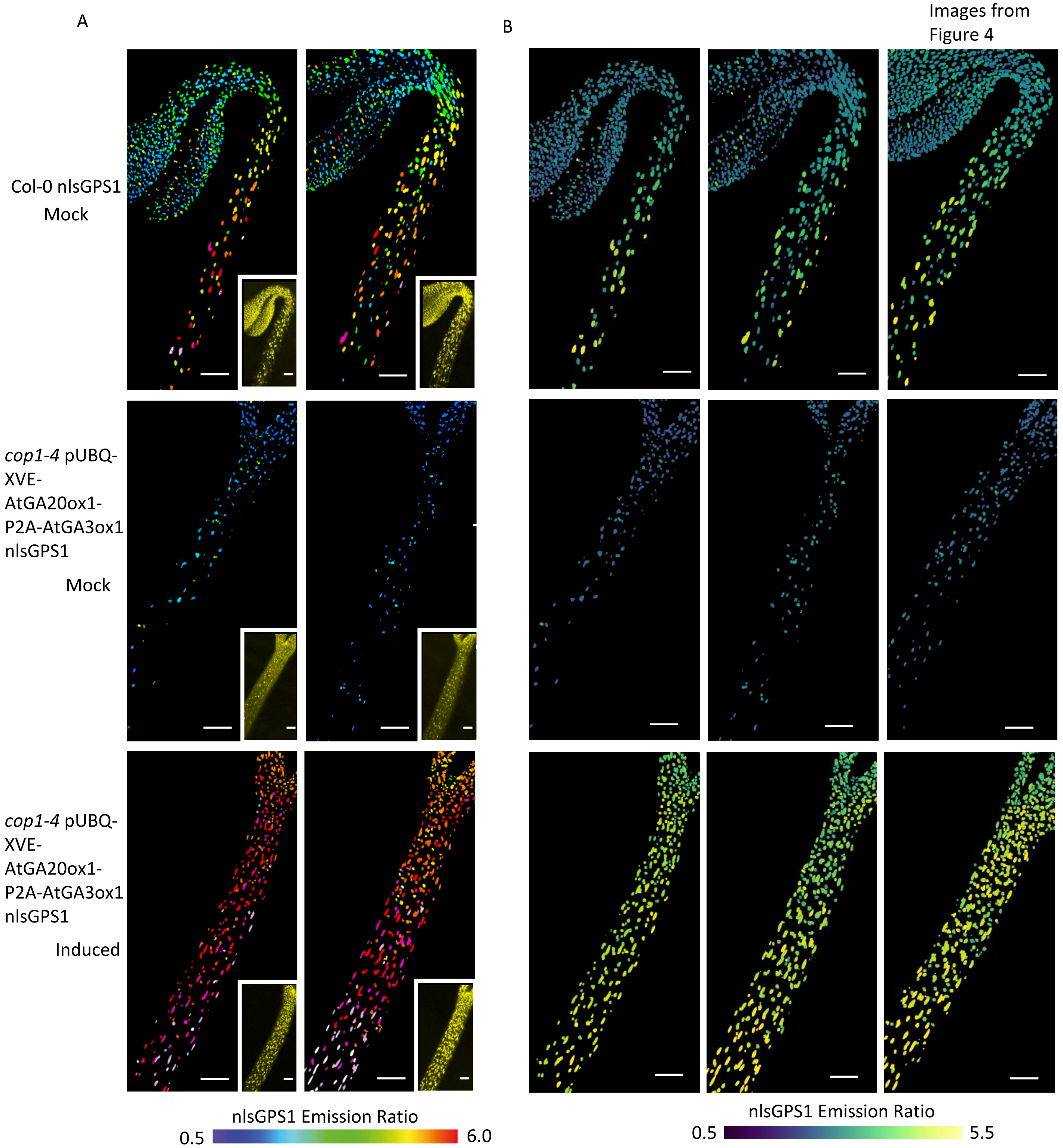

Supplementary Figure S11. Additional images of GA enzyme induction in cop1-4 nlsGPS1. (Supports Figure 4g and h). nlsGPS1 emission ratios of 3 day old dark grown hypocotyls β-estradiol inducible GA enzyme transgenic lines 48 h after induction with 2.5 µM 17-β-estradiol (induced) or with 0.1% DMSO mock induction (mock). A) 16_colors LUT. Representative images of emission ratios and YFP fluorescence (Inset) are shown. B) Color vision deficiency compatible LUT (Viridis) nuclei dilated (size increased) after analysis to allow easier visual discrimination of this LUT. (Images corresponding to emission ratio images presented in Figure 4g and h; Supplementary figure S12). Scale bar = 100µm

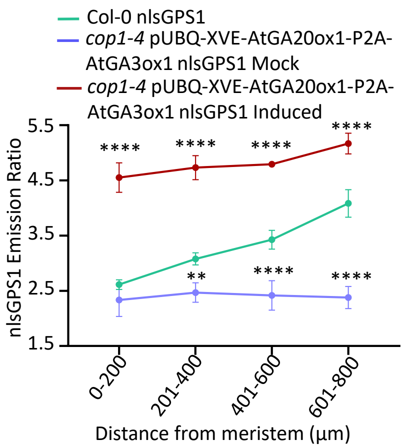

Supplementary Figure S12. Emission ratio of nlsGPS1 in Col-0 and mock treated and induced cop1-4 pUBQ-XVE-AtGA20ox1-P2A-AtGA3ox1 nlsGPS1. (Supports Figure 4). Mean of mean nuclear emission ratios from 3 hypocotyls, binning by distance from the shoot apical meristem of 3d dark grown hypocotyls of WT Col-0, β-estradiol inducible cop1-4 pUBQ-XVE-AtGA20ox1-P2A-AtGA3ox1 nlsGPS1 mock and induced. Error bars = SD Two-way ANOVA with a Dunnett's post hoc test was used for multiple comparisons comparing to Col-0 control. **p-value < 0.01 ****p-value <0.0001

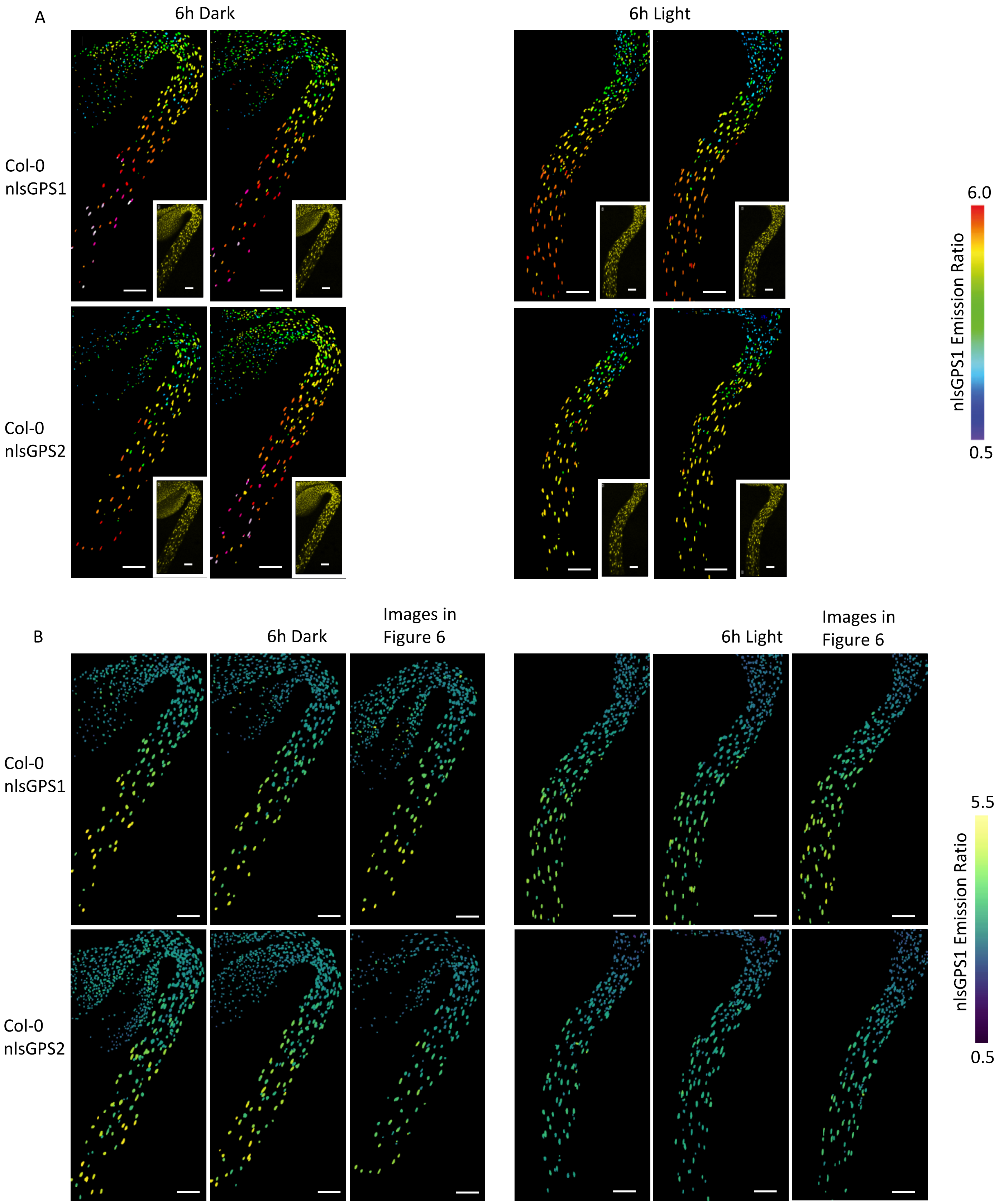

Supplementary Figure S13. Additional images of Dark to light transition Col-0 nlsGPS1 vs Col-0 nlsGPS2. (Supports Figure 6a and b). nlsGPS1 emission ratios of 3 day old dark grown hypocotyls 6h dark control and 6h after transfer to light. A) 16_colors LUT. Representative images of emission ratios and YFP fluorescence (Inset) are shown. B) Color vision deficiency compatible LUT (Viridis) nuclei dilated (size increased) after analysis to allow easier visual discrimination of this LUT. (Images corresponding to emission ratio images presented in Figure 6a and b). Scale bar = 100µm

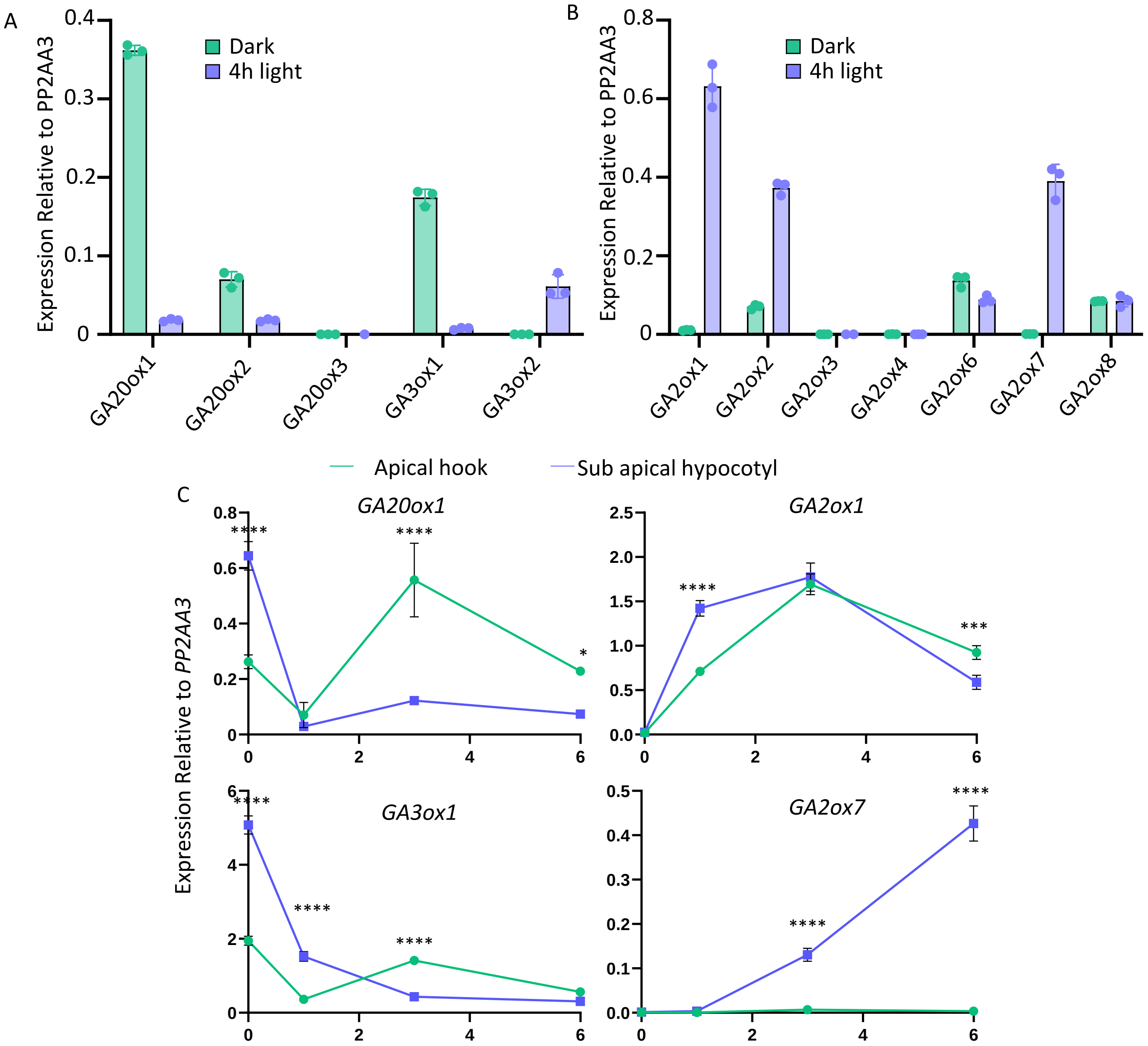

Figure S14. Gene expression in hypocotyls after transfer to light. (Supports Figure 6c). A) GA biosynthetic gene expression expressed relative to control gene PP2AA3 in dark grown hypocotyl and dark grown hypocotyl transferred to light for 4 hours. Error bars = SD B) GA catabolic gene expression expressed relative to control gene PP2AA3 in dark grown hypocotyl and dark grown hypocotyl transferred to light for 4 hours. Error bars = SD C) Independent replicate of relative expression changes of selected GA metabolic enzymes in the hook and sub-apical hook of Col-0 during de-etiolation shown in figure 6c. Dissections were carried out in the dark and at 1h, 3h and 6h light exposure. Data represents mean and standard deviation of three technical repeats from one biological repeat containing (30) dissected hypocotyls per sample. Error bars = SD Two-way ANOVA with a Šídák's multiple comparisons post hoc test *p-value < 0.05, ***p-value < 0.001, ****p-value < 0.0001

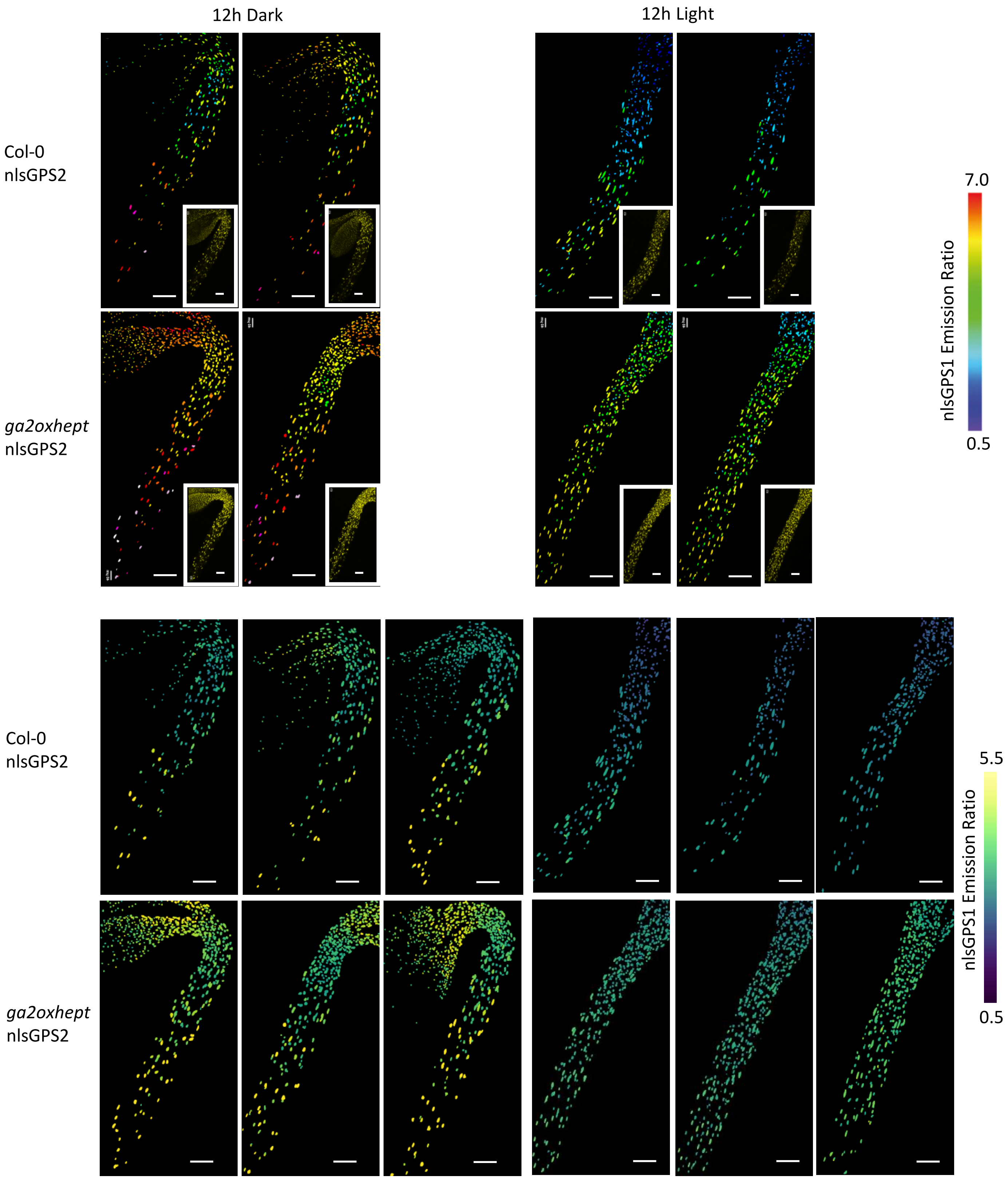

Supplementary Figure S15. Additional images of Dark to light transition Col-0 nlsGPS2 vs *ga2oxhept* nlsGPS2. (Supports Figure 6e and f). nlsGPS1 emission ratios of 3 day old dark grown hypocotyls 12h dark control and 12h after transfer to light. A) 16_colors LUT. Representative images of emission ratios and YFP fluorescence (Inset) are shown. B) Color vision deficiency compatible LUT (Viridis) nuclei dilated (size increased) after analysis to allow easier visual discrimination of this LUT. (Images corresponding to emission ratio images presented in Figure 6e and f). Scale bars = 100 µm

Primer Name	Gene ID	Gene Name	Purpose	Primer sequence 5'-3'	Pair with
PP2AA3_F	At1g13320	PP2AA3	RT-qPCR	AGACAAGGTTCACTCAATCCGTG	
PP2AA3_R	At1g13320	PP2AA3	RT-qPCR	CATTCAGGACCAAACTCTTCAGC	
GA3ox1_F	AT1G15550	GA3ox1	RT-qPCR	CCATTCACCTCCCACACTCT	
GA3ox1_R	AT1G15550	GA3ox1	RT-qPCR	GCCAGTGATGGTGAAACCTT	
GA3ox2_F	At1g80340	GA3ox2	RT-qPCR	TGGTCCGAAGGTTTCAC	
GA3ox2_R	At1g80341	GA3ox2	RT-qPCR	GGGTCGAGTCTGTATGG	
GA20ox1_F	AT4G25420	GA20ox1	RT-qPCR	GATCCATCCTCCACTTTAGA	
GA20ox1_R	AT4G25420	GA20ox1	RT-qPCR	GTGTATTCATGAGCGTCTGA	
GA20ox2_F	AT5G51810	GA20ox2	RT-qPCR	ACCGAGACTATTTCCGAGGATT	
GA20ox2_R	AT4G25420	GA20ox2	RT-qPCR	TGTTTGGCATGGAGGATAATG	
GA2ox1_F	AT1G78440	GA2ox1	RT-qPCR	TGAGGACGAGAGGTTGTACGA	
GA2ox1_R	AT1G78440	GA2ox1	RT-qPCR	TCCTTTCGAATTGTTGAAGCC	
GA2ox2_F	AT1G30040	GA2ox2	RT-qPCR	CCGGTTCTCACTTCCCATT	
GA2ox2_R	AT1G30040	GA2ox2	RT-qPCR	GCTTCCGGATCGGCTAG	
GA2ox4_F	AT1G47990	GA2ox4	RT-qPCR	GGCTCCAAGTGTCCAATTCA	
GA2ox4_R	AT1G47990	GA2ox4	RT-qPCR	TCCTACATTGACGCAGAAAGC	
GA2ox6_F	AT1G02400	GA2ox6	RT-qPCR	GGGACAGAAGTCTAGCGAAGTG	
GA2ox6_R	AT1G02400	GA2ox6	RT-qPCR	TCGCTACGAACGTCTCTGATC	
GA2ox7_F	At1g50960	GA2ox7	RT-qPCR	AGTAATGGAGTGTACCAAAGCG	
GA2ox7_R	At1g50960	GA2ox7	RT-qPCR	GAAAGCTATTGACATCCTCTCG	
cop1-4_F	AT2G32950	COP1	Genotyping	AATGCCGTTGAGAGACATAGAA	
cop1-4_R	AT2G32950	COP1	Genotyping	TGCCGGATTTCTGCTATAACTCT	
hy5-251_F	AT5G11260	HY5	Genotyping	GGCTCACCTTTCTCCACGAT	
hy5-251_R_WT	AT5G11260	HY5	Genotyping	TCTCCTCATCGCTTTCAATTCC	
hy5-251_R_hy5	AT5G11260	HY5	Genotyping	TCTCCTCATCGCTTTCAATTCT	
ga2ox1-1_F	AT1G78440	GA2ox1	Genotyping	TCTTCCGGTTCGATATCTCCCA	p745
ga2ox1-1_R	AT1G78440	GA2ox1	Genotyping	TGACCAAAACACGGACTCGAT	
ga2ox2-1_F	AT1G30040	GA2ox2	Genotyping	CTGCGAGGAGTTCGGGTTCTT	
ga2ox2-1_R	AT1G30040	GA2ox2	Genotyping	TTTTTGTCGACCCTCCACACC	LB1.3
ga2ox3-1_F	AT2G34555	GA2ox3	Genotyping	TTGAAAATTTGTCCTTTAACCCCA	
ga2ox3-1_R	AT2G34555	GA2ox3	Genotyping	TCCGGATGTGAAAACTGAAATCAA	LB1.3
ga2ox4-1_F	AT1G47990	GA2ox4	Genotyping	TGACAGCTCGGCAGTGAATTG	
ga2ox4-1_R	AT1G47990	GA2ox4	Genotyping	TGGGGTATCACATTTCACCTCAA	LB1.3
ga2ox6_2_F	AT1G02400	GA2ox6	Genotyping	TTGTCAACCGTATGGAAACCG	
ga2ox6_2_R	AT1G02400	GA2ox6	Genotyping	CAACCAAGAACCAACGATTGC	SLAT
ga2ox7-2_F	AT1G50960	GA2ox7	Genotyping	AGTCACCATGGACTTTCGC	
ga2ox7-2_R	AT1G50960	GA2ox7	Genotyping	GAGAAGTGGCGCTAGGGTTT	LB1.3
GA2ox8_F	AT4G21200	GA2ox8	Genotyping	AACGTTCGCAGACTCGTAGT	
GA2ox8_R	AT4G21200	GA2ox8	Genotyping	ACCTTTCTTTTGGTTAAGTTACCTT	LB1.3

Supplementary Table S1. Primers used in this study
