# Supplementary material for: GIBBERELLIN PERCEPTION SENSOR 2 reveals genesis and role of cellular GA dynamics in light-regulated hypocotyl growth
Source: Plant Cell. 2024 Jul 23;36(10):4426–41. doi: 10.1093/plcell/koae198 (PMC11449061; doi:10.1093/plcell/koae198)
Supplement: koae198_Supplementary_Data [file tpc.00366.2024-s06.pdf › tpc.00366.2024-s06.pdf]

THE PLANT CELL

Gibberellin Perception Sensors 1 and 2 reveal cellular GA dynamics articulated by COP1 and GA20ox1 that are necessary but not sufficient to pattern hypocotyl cell elongation

Jayne Griffiths, Annalisa Rizza, Bijun Tang, Wolf Frommer, and Alexander Jones

Review Timeline:

Submission Date:	06-Nov-2023
Editorial Decision:	16-Dec-2023
Resubmission Received:	23-Apr-2024
Accepted:	16-May-2024

Dr. Alexander M Jones
Sainsbury Laboratory
Cambridge, CB2 1LR
United Kingdom

Dear Dr. Jones:

Thank you for choosing to send your manuscript entitled "Gibberellin Perception Sensors 1 and 2 reveal cellular GA dynamics articulated by COP1 and GA20ox1 that are necessary but not sufficient to pattern hypocotyl cell elongation" for consideration at The Plant Cell. Your submission has been evaluated by members of the editorial board, as well as expert reviewers in your field. We note that these experts were also given access to the companion manuscript by Drake et al., however they were asked to review only this manuscript.

We regret to inform you that we are not able to recommend the publication of this manuscript in its current form. We have had input from multiple scientists and we have solicited post-review comments.

During the post-review consultation session, we also agreed that if you could address the major points raised by the reviewers by new experiments, we would welcome a resubmission. This may be treated as a new submission, but we would attempt to use at least some of the same reviewers.

As detailed in the reviews below, reviewers pointed out the need to address the role of HY5 gene in skotomorphogenesis and GA patterning, and the need to address the contribution of GA3ox1. Further, in the post-review consultation, there was unanimous agreement on the need to better explain the importance of the GPS2 sensor in the context of the current manuscript.

Please remember that if you decide to resubmit, reviewers will be asked to assess as a new manuscript, and not only whether previous reviewer comments have been addressed. It will be important to convince the editors and reviewers that the study adds significant new understanding(s) of mechanisms or processes, or otherwise represents a significant advance in the field, and that the major claims made are fully justified by the data presented.

The Plant Cell now requires authors to complete and submit an author revisions checklist upon submission of a revised manuscript. The aim of the checklist is to aid authors in preparing a high-quality manuscript, facilitate the review and assessment of revised manuscripts, and help to ensure that journal standards are maintained across the board. If your manuscript is accepted, the completed checklist will be published as supplemental material attached to the article online. Please download a copy of the checklist (a fillable PDF form) at this link, for submission with your revised manuscript: https://tpc.msubmit.net/html/Author_Revisions_Checklist.pdf.

Note also that supplemental materials should be restricted to large datasets and tables, presentation of replicates, and validation of reagents, methods, or genotypes. Any data that are used to support the main claims must be in the main manuscript. Supplemental figure legends must indicate what figure in the main manuscript is supported by the supplemental data presented.

We thank you for your interest in and support of The Plant Cell. We hope we will be able to render a more positive decision on future work.

Sincerely,

The Plant Cell Editorial Board

-- Reviewer comments:

Reviewer #1 (Comments for the Author):

The study of Griffiths et al. contains some very interesting data on skotomorphogenesis and the role of GA patterning and biosynthesis in this process. First of all, they show through time-lapse imaging that pifq immediately progresses to cotelydon opening once the apical hook has been set and that this coincides perfectly with a loss of the GA gradient as perceived with GPS1. They show that even in white light, the phyabcry12 mutant establishes a GA gradient. Interestingly, the cop1-4 mutant does not have a GA gradient, and important GA biosynthesis genes are misexpressed in cop1-4. Adding the hy5 mutation to cop1 does not rescue the gradient, however it does partially rescue GA20ox1 expression, which the authors identify later as

being the primary driver of the gradient, by ectopically inducing it. This induction results in a general increase of GA, and the loss of a GA gradient. Surprisingly, GA3ox1 induction does not have any effect on GA levels as measured by GPS1 at all. Then it is shown that the establishment of a GA gradient is not related to cell length patterning, a very interesting finding! The authors then went through the trouble of creating a septuple GA2ox mutant through classical crossing (wow!), which has a slight defect in the GA gradient. Finally, they show that the loss of the GA gradient in cop1-4 can be rescued via induction of GA biosynthesis through GA20ox1. But funnily enough, the correlation between cell length and GPS1 ratio in cop1-4 cannot be restored by inducing GA20ox1, showing again that GA does not set the cell length.

Then we get to part 2 of the paper, which deals with photomorphogenesis and a new GPS1 sensor. I think the construction of this sensor is great, and it's extremely laudable how the authors strive to improve their FRET sensors. However, as a story for this paper, it's completely disconnected from the first part, since the final figure, figure 6, deals with the dark-to light transition. It is shown that GPS2 can detect the GA changes upon photomorphogenesis and that GA biosynthesis and catabolism genes are changed in expression. However, this has not much to do with the previous part of the manuscript, which deals with skotomorphogenesis and does not use the GPS2 sensor at all!

Thus, my biggest problem with this manuscript is that it is two papers, not one. If one finishes the manuscript after Figure 4, we have some really interesting data, that do need some extra clarification.

In my opinion there are two important analyses missing:

- hy5 GPS1nls analysis
The authors should include an analysis of the GPS1 sensor in the hy5 mutant alone to go with figure2. I am wondering whether the hy5 mutant still has a GA gradient. To simply disregard this mutant with the argument that hy5 activity in the dark is low is not enough.

- proof that GA3ox1 is actually induced, perhaps through qPCR
Since GA3ox1 induction has no effect at all, we are left wondering whether it induces effectively at all. Some proof of expression is needed.

Furthermore, it would have been good to see GA20ox1 induction in the ga20ox1(,2,3) mutant, to compare it against induction of GA20ox1 in the Col-0 wild type background.

Then there are some smaller and some textual remarks:

Fig. 2: Is there data about the hook angle of cop1 and cop1hy5? It would be nice to have that with this figure, even though the result is kind of obvious.
fig. 3c: It is not clear to which thing the expression changes are relative to.

Fig. 3d: It is not well explained how the double induction of ga20ox1 and ga3ox1 works. Are they fused together as a construct? it is not well explained how the inducible constructs are made in the methods

line 270: If GA20ox1 is the primary driver of the gradient, why then don't we see the gradient in cop1 hy5 where the GA20ox expression is back to normal?
It would be good if this features in the discussion

Fig. 4c is not labeled

Fig. 4g-I So indeed, COP1 creates the gradient, but not just via hy5

line 194: we created a phyAphyB..

line 219: Interestingly... GA3ox2 is expression differences are also rescued by hy5 mutation. Why is this not mentioned? GA3ox2 seems to be completely ignored in the text, even though the data shows interesting differences with this gene in the cop1 mutant. Now it is clear, also from the dark-to-light transition, that GA3ox2 is associated with photomorphogenesis. Maybe it's good to mention that.

line 460: "neither.." what is exactly meant with this sentence?

line 470: "The selected transcripts examined were those that demonstrated the greatest fold change in the whole hypocotyl at 4 hour transfer to light (Figure S11a)"
This seems to be a false statement, since GA3ox2 and not Ga3ox1 had the highest fold change after 4 hrs. Why have the authors not selected GA3ox2 then?

Line 478: "In contrast, GA2ox7 expression is strongly increased but this is limited to the apical hook region (Figure 6c)." This should be the sub-apical hypocotyl!

line 535: "COP1 signalling acting, presumably indirectly, on the patterned expression of the GA20ox1 biosynthetic enzyme is a central regulatory module"
I don't think the authors have showed it acts on the patterned expression. It has an effect on the expression in the whole hypocotyl, mostly via HY5.

Fig. S4: final graph does not have a title.

Reviewer #3 (Comments for the Author):

Please, find enclosed the reviewing of the following manuscript: TPC2023-RA-01051;
Entitled: "Gibberellin Perception Sensors 1 and 2 reveal cellular GA dynamics articulated by COP1 and GA20ox1 that are necessary but not sufficient to pattern hypocotyl cell elongation" by Jayne Griffiths, Annalisa Rizza, Bijun Tang, Wolf B. Frommer, Alexander M. Jones.

In this manuscript, Griffiths et al. have sought to understand how light signalling integrates into cellular GA regulation on Arabidopsis hypocotyls, during deetiolation process, as referring to the shift of the skotomorphogenesis program of elongating hypocotyls, in contrast, to the resetting of restrain hypocotyl elongation upon photomorphogenesis.
One of the major interests of the study relies on the engineered tool to decipher tissue/cell GA dynamic during this deetiolation event. Indeed, to further decipher the GA dynamic, the authors have engineered a second generation GPS2 biosensor (nuclear localized Gibberellin Perception Sensor 2-nlsGPS2, based on FRET technology) with improved reversibility to show the cellular pattern of GA depletion during the transition to growth in the light.

In this manuscript, the authors have combined biosensing, chemical, genetic and transcriptomic (more precisely RT-qPCR) analysis, to partially unravel which steps are patterned to articulate cellular GA dynamics in the hypocotyl during deetiolation process, and how they are themselves regulated, among the wide inner regulatory network and outer light/darkness inputs.

Overall, the experimental setting sequences and provided results are progressive didactic, robust and clear.
- 'Skotomorphogenesis agonist PIFs' (required to maintain, but not generate GA gradient in dark-grown hypocotyls). The results are convincing and authors propose adequate conclusions.
- 'Brassinosteroid distribution does not explain GA gradient in dark-grown hypocotyls. The results are convincing and authors mentioned adequate developed conclusions. The figures 1 and S1, S2 are well presented, legend complete, except may be, to add a bar scale in the photos of the pifq mutant seedlings grown from 43h to 53h (fig.1a), as I would also suggest to had it, in figures 3a and 4a. Statistical analysis is precise and robust.
- 'Skotomorphogenesis agonist COP1 is required for generating GA gradient'
In the context of the known light signal cascade through photoreceptors PHYs and CRYs, and key players COP1 E3 ligase and its primary target, the bZIP transcription factor ELONGATED HYPOCOTYL 5 (HY5) - a positive regulator of photomorphogenesis, the authors have generated diverse nlsGPS1 biosensor lines in multiple mutant genetic backgrounds, to reveal mechanisms which potentially contribute to establish GA gradient within different hypocotyl sections, during growth in dark or light transition exposure. All data shown in figures 2 and S2 with biosensor imaging and data assembled in graphs are relevant and well presented. Moreover, the combined RT-qPCR experiments on genes involved in biosynthesis and catabolism, highlights the involvement of GA20ox1in the patterning of GA, as primary target of COP1-HY5 regulatory module.
Note: I would suggest that the authors rather write RT-qPCR, in line 215 (not only qPCR), as well mentioned in line 284.
- The GUS-reporter lines have shown that GA20ox1 and GA3ox1 expressions are correlated with GA gradient but only GA20ox1 expression seems rate limiting in dark-grown hypocotyls, which could be correlated with the fact that GA3ox1 seemed not to be primary target of Hy5, as mentioned by the authors.
- 'GA catabolism also plays a role in generating the GA gradient in dark-grown hypocotyls, as shown in the figure 4d and e, at least in the hypocotyl section closed to the meristem. However, more interestingly, 'abolishing the GA gradient is not sufficient to abolish cell length patterning.'
The authors have demonstrated that GA biosynthesis plays a key role in the formation of the apical-basal gradient emphasizing that GA20ox1 and GA3ox1 represent key targets of the COP1 pathway. Noteworthy, an interesting result/observation is shown in the experiment depicted in (Figure 4i), in which in the cop1-4 background, both mock and β-estradiol induced pUBQ-XVE-AtGA20ox1-P2A-AtGA3ox1 hypocotyl cells exhibited cell length patterning without correlation with cellular GA levels.

As specified by the authors, 'although GPS1biosensor has high affinity to bioactive GA and reveals cellular distribution and regulation of GA, nevertheless its slow reversibility precluded use for investigation of GA depletion dynamics.' Thus, the authors

have engineered a second-generation Gibberellin Perception Sensor 2 (GPS2), which seems to improve reversibility (as shown in figure 5e), and orthogonality, by introducing specific charge exchange mutations (at E13K and E28K (AtGID1C) and K51E and R54E (AtGAI) into GPS1).
All the data shown are reliable for depicting this new generation of biosensor nlsGPS2, nonetheless, could the authors specify more the argument for which this biosensor has improved its orthogonality. I do believe that this sensor is an improved tool to monitor GA gradient dynamic patterning, as shown in the comparative assay illustrated in figure 6b, then able to detect a depletion of hypocotyl GA (not detected with nlsGPS1 biosensor). But still, I miss some explanation about its orthogonality property. However, this result is undoubtedly of interest. The results provided in the figure 6a and b are rather convincing and confirm to fit better with previous reports. Moreover, the experimental design related to the dark to light time course, figure 6c, and the obtained results (with nlsGPS2 imaging and in parallel, RT-qPCR on GA biosynthesis or catabolic genes, allow with accuracy to decipher local and transient (dynamic) cellular GA content to pattern GA gradient, which contributes to cell growth regulation (extension or slowing elongation) of hypocotyls during dark to light transition. Data of fine tune regulation of GA biosynthesis or catabolic genes, in apical hook and sub-apical region of hypocotyls, are outstanding and reflect a pinpoint accuracy.

From this work, the authors have nicely shown/demonstrated that 'upon transfer to light, reduced biosynthesis and increased catabolism in the hypocotyl leads to decreased GA levels and this depletion is quantitatively important for slowing elongation' in hypocotyls; emphasizing the fact that local or temporal GA control is meaningful in this specific plastic trait, among others already established. With nlsGPS2 biosensor, the authors have opened an area of investigation in which in vivo study allows to quantify the relationship between environmentally sensitive cellular GA dynamics and developmental plasticity.
Altogether, the content of this paper is undoubtedly of interest, completes results initiated by the researchers in 2017 and 202, with meaningful innovative results.

We are grateful to the reviewers for their helpful critiques and suggestions as well as many positive comments. In this revision, we have addressed all comments in a response to reviewers document and made improvements to the manuscript including additional or improved figure panels and text changes highlighted in blue.

The main changes include:

- Introduction of a synchronised seed position during germination for improved characterisation of *pifq* mutant etiolated development
- Reduced variation in a more thorough characterization of BR crosstalk with GA
- Inclusion of *hy5* GPS1 data shows HY5 has no effect in the dark
- Inclusion of *cop1* and *cop1/hy5* hook angle in comparison to *pifq* demonstrates the distinct development of these lines
- Discussion of *GA3ox2* changes in *cop1*/de-etiolation to acknowledge the discrepancy with overall GA levels
- Addition of a cell size vs cell position analysis in our *cop1* GA complementation lines
- Addition of *ga2oxheptuple* nlsGPS2 line analysed during de-etiolation to demonstrate the importance of reducing GA for growth reprogramming
- Additional points of clarification and discussion throughout as suggested by referees.

Reviewer #1 (Comments for the Author):

The study of Griffiths et al. contains some very interesting data on skotomorphogenesis and the role of GA patterning and biosynthesis in this process. First of all, they show through time-lapse imaging that pifq immediately progresses to cotelydon opening once the apical hook has been set and that this coincides perfectly with a loss of the GA gradient as perceived with GPS1. They show that even in white light, the phyabcry12 mutant establishes a GA gradient. Interestingly, the cop1-4 mutant does not have a GA gradient, and important GA biosynthesis genes are misexpressed in cop1-4. Adding the hy5 mutation to cop1 does not rescue the gradient, however it does partially rescue GA20ox1 expression, which the authors identify later as being the primary driver of the gradient, by ectopically inducing it. This induction results in a general increase of GA, and the loss of a GA gradient. Surprisingly, GA3ox1 induction does not have any effect on GA levels as measured by GPS1 at all. Then it is shown that the establishment of a GA gradient is not related to cell length patterning, a very interesting finding! The authors then went through the trouble of creating a septuple GA2ox mutant through classical crossing (wow!), which has a slight defect in the GA gradient. Finally, they show that the loss of the GA gradient in cop1-4 can be rescued via induction of GA biosynthesis through GA20ox1. But funnily enough, the correlation between cell length and GPS1 ratio in cop1-4 cannot be restored by inducing GA20ox1, showing again that GA does not set the cell length.

Then we get to part 2 of the paper, which deals with photomorphogenesis and a new GPS1 sensor. I think the construction of this sensor is great, and it's extremely laudable how the authors strive to improve their FRET sensors.

We thank the reviewer for these detailed comments and careful analysis of our work.

However, as a story for this paper, it's completely disconnected from the first part, since the final figure, figure 6, deals with the dark-to light transition. It is shown that GPS2 can detect

the GA changes upon photomorphogenesis and that GA biosynthesis and catabolism genes are changed in expression. However, this has not much to do with the previous part of the manuscript, which deals with skotomorphogenesis and does not use the GPS2 sensor at all!

Thus, my biggest problem with this manuscript is that it is two papers, not one.

We apologise that the need to engineer GPS2 to do the photomorphogenesis experiments properly is a subtlety that was not sufficiently explained. GPS1 is suitable for steady state distributions and GA increases in the first part of the manuscript and prior publications. The reversibility of GPS2 provides the ability to detect GA depletion dynamics in photomorphogenesis that GPS1 does not. There are dozens of novel transgenic lines supporting this study and most of these were made before GPS2 was engineered. Completing all analyses with the new sensor was not possible. Nonetheless, the zero time points and negative controls in the latter part of the manuscript do make use of GPS2's improved features in analysing skotomorphogenesis and the results were similar to GPS1 for steady state distributions and GA overaccumulations. The topical transition and its justification are now more clearly signposted and explained in the abstract, introduction, results and discussion.

If one finishes the manuscript after Figure 4, we have some really interesting data, that do need some extra clarification.

We thank the reviewer for many helpful suggestions and have addressed all comments below.

In my opinion there are two important analyses missing:

- hy5 GPS1nls analysis
The authors should include an analysis of the GPS1 sensor in the hy5 mutant alone to go with figure2. I am wondering whether the hy5 mutant still has a GA gradient. To simply disregard this mutant with the argument that hy5 activity in the dark is low is not enough.

This has been done and included in Figure 2e and f.

- proof that GA3ox1 is actually induced, perhaps through qPCR
Since GA3ox1 induction has no effect at all, we are left wondering whether it induces effectively at all. Some proof of expression is needed.

We used a published line (Rizza et al., 2021 https://doi.org/10.1073/pnas.1921960118 Figure S7A) for this work. We have now clarified this in the manuscript. Notably, in the root it is GA3ox expression and not GA20ox that is rate-limiting in elongating cells.

Furthermore, it would have been good to see GA20ox1 induction in the ga20ox1(,2,3) mutant, to compare it against induction of GA20ox1 in the Col-0 wild type background.

Whilst there are likely interesting results from this experiment regarding transition from the extremely low GA levels and associated developmental reprogramming of the *ga20ox1,2,3* triple mutant through to very high GA levels of the overexpression state, we consider these transgenic lines and experiments to be beyond the scope of this manuscript. This is to keep the present focus on regulation and function of the wild-type GA gradient in skotomorphogenesis and it's dynamics in photomorphogenesis.

Then there are some smaller and some textual remarks:

Fig. 2: Is there data about the hook angle of cop1 and cop1hy5? It would be nice to have that with this figure, even though the result is kind of obvious.

We now include data on hook angle at 26 hrs post germination (Supp Fig 5) showing that *cop1* and *cop1hy5* open rapidly whilst *pifq* and Col0 form hooks.

fig. 3c: It is not clear to which thing the expression changes are relative to.

This has been made clearer and the expression is now expressed relative to control gene PP2AA3.

Fig. 3d: It is not well explained how the double induction of ga20ox1 and ga3ox1 works. Are they fused together as a construct? it is not well explained how the inducible constructs are made in the methods

These constructs and lines were made and characterised in a previous publication (Rizza et al., 2021 https://doi.org/10.1073/pnas.1921960118) This is now more clearly explained and referenced in the manuscript.

line 270: If GA20ox1 is the primary driver of the gradient, why then don't we see the gradient in cop1 hy5 where the GA20ox expression is back to normal?
It would be good if this features in the discussion

We now explore this result in further depth in the results and discussion.

Fig. 4c is not labeled
Labels have been added.

Fig. 4g-I So indeed, COP1 creates the gradient, but not just via hy5
We agree that COP1 is required for GA20ox patterning and the GA gradient. HY5 is a repressor during photomorphogenesis, but does not control spatial patterning in darkness.

line 194: we created a phyAphyB..
This typo has been corrected.

line 219: Interestingly... GA3ox2 is expression differences are also rescued by hy5 mutation. Why is this not mentioned? GA3ox2 seems to be completely ignored in the text, even though the data shows interesting differences with this gene in the cop1 mutant. Now it is clear, also from the dark-to-light transition, that GA3ox2 is associated with photomorphogenesis. Maybe it's good to mention that.

Thank you for pointing out this omission. We have now included the interesting contrast between GA3ox1/GA20ox1 vs GA3ox2 dynamics in the results and included non-normalised expression data in Supp Fig 14.

line 460: "neither.." what is exactly meant with this sentence?

“Neither sensor reported any localised GA increases at this time point.” This sentence has been removed as it was referring to a later result, namely the increase of transcription of biosynthesis enzymes in the hook.

line 470: "The selected transcripts examined were those that demonstrated the greatest fold change in the whole hypocotyl at 4 hour transfer to light (Figure S11a)"
This seems to be a false statement, since GA3ox2 and not Ga3ox1 had the highest fold change after 4 hrs. Why have the authors not selected GA3ox2 then?

Apologies for this error. GA3ox2 was not selected as it is very low expressed in etiolated hypocotyls and is unlikely to explain the GA gradient or its dynamics. We have revised the manuscript to include discussion of GA3ox2 dynamics, corrected the erroneous statement and now present non-normalised data in Supp Fig 14b.

Line 478: "In contrast, GA2ox7 expression is strongly increased but this is limited to the apical hook region (Figure 6c)." This should be the sub-apical hypocotyl!

Thank you for spotting this error. It has been corrected.

line 535: "COP1 signalling acting, presumably indirectly, on the patterned expression of the GA20ox1 biosynthetic enzyme is a central regulatory module"
I don't think the authors have showed it acts on the patterned expression. It has an effect on the expression in the whole hypocotyl, mostly via HY5.

We previously stated that the link was likely indirect. The direct driver(s) of the GA20ox spatial expression pattern remain unknown and is the topic of a future study. We have now revised the statement to clarify that COP1 signalling could be permissive for unknown pattern forming agent(s). Notably, even with HY5 absent in the *cop1 hy5* mutant in darkness, the pattern forming agent(s) seem to be absent. In wild type, HY5 action is important in photomorphogenesis and GA depletion.

Fig. S4: final graph does not have a title.

Thank you for spotting this error. It has been corrected.

Reviewer #3 (Comments for the Author):

Please, find enclosed the reviewing of the following manuscript: TPC2023-RA-01051; Entitled: "Gibberellin Perception Sensors 1 and 2 reveal cellular GA dynamics articulated by COP1 and GA20ox1 that are necessary but not sufficient to pattern hypocotyl cell elongation" by Jayne Griffiths, Annalisa Rizza, Bijun Tang, Wolf B. Frommer, Alexander M. Jones.

In this manuscript, Griffiths et al. have sought to understand how light signalling integrates into cellular GA regulation on Arabidopsis hypocotyls, during deetiolation process, as referring to the shift of the skotomorphogenesis program of elongating hypocotyls, in contrast, to the resetting of restrain hypocotyl elongation upon photomorphogenesis.
One of the major interests of the study relies on the engineered tool to decipher tissue/cell GA

dynamic during this deetiolation event. Indeed, to further decipher the GA dynamic, the authors have engineered a second generation GPS2 biosensor (nuclear localized Gibberellin Perception Sensor 2-nlsGPS2, based on FRET technology) with improved reversibility to show the cellular pattern of GA depletion during the transition to growth in the light.

In this manuscript, the authors have combined biosensing, chemical, genetic and transcriptomic (more precisely RT-qPCR) analysis, to partially unravel which steps are patterned to articulate cellular GA dynamics in the hypocotyl during deetiolation process, and how they are themselves regulated, among the wide inner regulatory network and outer light/darkness inputs.

Overall, the experimental setting sequences and provided results are progressive didactic, robust and clear.

We thank the reviewer for these positive comments and for their very helpful suggestions.

- 'Skotomorphogenesis agonist PIFs' (required to maintain, but not generate GA gradient in dark-grown hypocotyls). The results are convincing and authors propose adequate conclusions.
- 'Brassinosteroid distribution does not explain GA gradient in dark-grown hypocotyls. The results are convincing and authors mentioned adequate developed conclusions. The figures 1 and S1, S2 are well presented, legend complete, except may be, to add a bar scale in the photos of the pifq mutant seedlings grown from 43h to 53h (fig.1a), as I would also suggest to had it, in figures 3a and 4a. Statistical analysis is precise and robust.
- 'Skotomorphogenesis agonist COP1 is required for generating GA gradient'
In the context of the known light signal cascade through photoreceptors PHYs and CRYs, and key players COP1 E3 ligase and its primary target, the bZIP transcription factor ELONGATED HYPOCOTYL 5 (HY5) - a positive regulator of photomorphogenesis, the authors have generated diverse nlsGPS1 biosensor lines in multiple mutant genetic backgrounds, to reveal mechanisms which potentially contribute to establish GA gradient within different hypocotyl sections, during growth in dark or light transition exposure. All data shown in figures 2 and S2 with biosensor imaging and data assembled in graphs are relevant and well presented. Moreover, the combined RT-qPCR experiments on genes involved in biosynthesis and catabolism, highlights the involvement of GA20ox1in the patterning of GA, as primary target of COP1-HY5 regulatory module.
Note: I would suggest that the authors rather write RT-qPCR, in line 215 (not only qPCR), as well mentioned in line 284.

Thank you for spotting this error. It has been corrected

- The GUS-reporter lines have shown that GA20ox1 and GA3ox1 expressions are correlated with GA gradient but only GA20ox1 expression seems rate limiting in dark-grown hypocotyls, which could be correlated with the fact that GA3ox1 seemed not to be primary target of Hy5, as mentioned by the authors.
- 'GA catabolism also plays a role in generating the GA gradient in dark-grown hypocotyls, as shown in the figure 4d and e, at least in the hypocotyl section closed to the meristem. However, more interestingly, 'abolishing the GA gradient is not sufficient to abolish cell length patterning.'
The authors have demonstrated that GA biosynthesis plays a key role in the formation of the apical-basal gradient emphasizing that GA20ox1 and GA3ox1 represent key targets of the

COP1 pathway. Noteworthy, an interesting result/observation is shown in the experiment depicted in (Figure 4i), in which in the cop1-4 background, both mock and β-estradiol induced pUBQ-XVE-AtGA20ox1-P2A-AtGA3ox1 hypocotyl cells exhibited cell length patterning without correlation with cellular GA levels.

We have now included another analysis of this dataset (see Fig 4 and Supp Fig 12) for another view of the phenomenon (plotting ‘cell length vs distance from meristem’ in addition to ‘nlsGPS1 emission ratio vs cell length’).

As specified by the authors, 'although GPS1biosensor has high affinity to bioactive GA and reveals cellular distribution and regulation of GA, nevertheless its slow reversibility precluded use for investigation of GA depletion dynamics.' Thus, the authors have engineered a second-generation Gibberellin Perception Sensor 2 (GPS2), which seems to improve reversibility (as shown in figure 5e), and orthogonality, by introducing specific charge exchange mutations (at E13K and E28K (AtGID1C) and K51E and R54E (AtGAI) into GPS1).
All the data shown are reliable for depicting this new generation of biosensor nlsGPS2, nonetheless, could the authors specify more the argument for which this biosensor has improved its orthogonality. I do believe that this sensor is an improved tool to monitor GA gradient dynamic patterning, as shown in the comparative assay illustrated in figure 6b, then able to detect a depletion of hypocotyl GA (not detected with nlsGPS1 biosensor). But still, I miss some explanation about its orthogonality property. However, this result is undoubtedly of interest.

The orthogonality properties are designed in the new sensor via the charge-exchange mutations that allow intra-molecular interaction within the sensor while disrupting inter-molecular interactions with endogenous GID1 and DELLA proteins. The test of this new feature is found in Figure 5G where expression of nlsGPS2 does not affect sensitivity to a GA biosynthesis inhibitor whilst nlsGPS1 does. This key improvement in GPS2 function for *in planta* studies has now been clarified in the manuscript.

The results provided in the figure 6a and b are rather convincing and confirm to fit better with previous reports. Moreover, the experimental design related to the dark to light time course, figure 6c, and the obtained results (with nlsGPS2 imaging and in parallel, RT-qPCR on GA biosynthesis or catabolic genes, allow with accuracy to decipher local and transient (dynamic) cellular GA content to pattern GA gradient, which contributes to cell growth regulation (extension or slowing elongation) of hypocotyls during dark to light transition. Data of fine tune regulation of GA biosynthesis or catabolic genes, in apical hook and sub-apical region of hypocotyls, are outstanding and reflect a pinpoint accuracy.

From this work, the authors have nicely shown/demonstrated that 'upon transfer to light, reduced biosynthesis and increased catabolism in the hypocotyl leads to decreased GA levels and this depletion is quantitatively important for slowing elongation' in hypocotyls; emphasizing the fact that local or temporal GA control is meaningful in this specific plastic trait, among others already established. With nlsGPS2 biosensor, the authors have opened an area of investigation in which in vivo study allows to quantify the relationship between environmentally sensitive cellular GA dynamics and developmental plasticity.
Altogether, the content of this paper is undoubtedly of interest, completes results initiated by the researchers in 2017 and 202, with meaningful innovative results.

We thank the reviewer for the positive comments regarding the quantitative analysis of cellular GA levels and dynamics. We have added additional detailed experiment to compare GA depletion in wild type vs the *ga2ox heptuple* mutant during photomorphogenesis (Fig 6 e & f). As expected, the mutant GA levels start higher and fall more slowly and this quantitative view can now be more directly linked with the modest overextension phenotype previously shown in Fig 6.

Dear Dr. Alexander Jones:

We are pleased to inform you that your paper entitled "Gibberellin Perception Sensors 1 and 2 reveal cellular GA dynamics articulated by COP1 and GA20ox1 that are necessary but not sufficient to pattern hypocotyl cell elongation" has been accepted for publication in The Plant Cell, pending a final editorial review by a science editor. At this stage, your manuscript will be evaluated by a science editor with respect to its presentation of scientific content, compliance with journal policies, and presentation for a broad readership. The Plant Cell has contracted with Plant Editors (planteditors.com) to provide this service to our authors, and you will soon receive additional information on this process.

Please note that each author needs to link their ORCID identifier to their account in the system before your manuscript can be published. If any authors do not have an ORCID linked to their account, they will receive a message with a link to complete this task. Please ensure that ALL of your coauthors have completed this task as soon as possible.

ASPB offers an OPEN option that allows authors to have their online articles available for free to all users immediately upon publication. For more information about the ASPB OPEN option, refer to the Final Submission Checklist Form.

The Plant Cell and The Arabidopsis Information Resource (TAIR) are collaborating to collect functional annotation data about Arabidopsis genes from authors. This includes information about the gene's molecular function (e.g., kinase activity, ATP synthetase activity), the biological process/es it is involved in (e.g., endosperm development, threonine biosynthesis), its subcellular location (e.g., nucleus, ER), anatomical or developmental expression pattern (e.g., leaf, ovule, flower stage 10, seedling stage), or its partner in a protein-protein interaction (e.g., AT1G01010 interacts with AT1G01020).

If your paper contains results falling into one or more of these categories for Arabidopsis genes, we request that you now submit these data for inclusion in TAIR by filling in the form provided at the following URL: https://www.arabidopsis.org/doc/submit/functional_annotation/123. If you need further clarification on what types of data can be submitted please contact curator@arabidopsis.org.

Finally, we encourage your submission of artwork for the journal cover. Monthly issues will have an online cover image and selected covers will be used for posters, other promotional items, and "wallpaper" for mobile devices. For more information, refer to Cover Submission in the Instructions for Authors https://tpc.msubmit.net/cgi-bin/main.plex?form_type=display_auth_instructions.

We look forward to seeing your paper published.

Sincerely,

The Plant Cell Editorial Board

=====
IMPORTANT REMINDER: PEER REVIEW REPORTS
=====

If you opted to publish a peer review report along with your article during the original submission process, it will be prepared by the editorial staff and publicly posted with your manuscript, inside the zip file that contains any other supplemental material. As a reminder, the peer review report is a public record of all comments from editors and reviewers, as well as your prior responses, as you received them in the decision letters for each draft of your manuscript. If you agreed to publish this report and have changed your mind, or are not sure if you selected this option, please contact the editorial office as soon as possible before signing the license agreement from our publisher.

=====
REVIEWER COMMENTS
=====

Reviewer #1 (Comments for the Author):

Dear authors, I am satisfied with the improvements made to the manuscript. I think it's clearer now and some obvious things that were missing have been adressed.

Reviewer #3 (Comments for the Author):

Please, find enclosed the second reviewing of the following manuscript: TPC2024-RA-00 366D;
Entitled: "Gibberellin Perception Sensors 1 and 2 reveal cellular GA dynamics articulated by COP1 and GA20ox1 that are necessary but not sufficient to pattern hypocotyl cell elongation" by Jayne Griffiths, Annalisa Rizza, Bijun Tang, Wolf B. Frommer, Alexander M. Jones.

In the revised manuscript, and the answers to the comments made by the reviewers, the authors have clarified and provided substantially new data to depict the skotomorphogenesis process, and the role of GA patterning and biosynthesis during deetiolation process, and partially revealed how they are themselves regulated.
All additive informations are clear, relevant. The answers provided by the authors are concise and convincing, to me.

Moreover, I particularly appreciate the new and relevant data, like 'Addition of a cell size vs cell position analysis in the cop1 GA complementation lines (Fig 4 and Supp Fig 12); And the addition of ga2oxheptuple nlsGPS2 line analyzed during de-etiolation to correlate (and demonstrate) the importance of reducing GA for growth reprogramming, (Fig 6 e & f; Fig S15). New data shown in the Figure S14 are clear and interesting, in particular, part c, in apical hook (relative to sub apical hypocotyl) for GA20ox1 and less extend GA3ox1, then for GA2ox7. Then, I agree with the authors conclusion that "differential spatiotemporal expression of GA20ox1, GA3ox1 and GA2ox7 could explain the differential extent of GA depletion along the growth axis observed with nlsGPS2 (Figure 6)".

It has been a pleasure to review this scientific manuscript, with well-designed experiments and performant tool (GPS2 biosensor (nuclear localized Gibberellin Perception Sensor 2-nlsGPS2), providing interesting data, which imply supports to new understandings of how GA patterning and biosynthesis and their regulation, contribute to the resetting of restrain hypocotyl elongation upon photomorphogenesis (de-etiolation) and the changes in the apical hook opening.
Best regards

----FOR ASPB OFFICE USE ONLY (DO NOT EDIT)----
MSID: 39124
Corresponding Author: Dr. Jones
